# Implementation barriers and facilitators to a vocational rehabilitation intervention after traumatic injury (ROWTATE) in the UK: qualitative interviews with key stakeholders

**DOI:** 10.1136/bmjopen-2026-118198

**Published:** 2026-07-30

**Authors:** Claire Mann, Rebecca Lindley, Denise Kendrick, Kate Radford, Jain Holmes, Blerina Kellezi, Roshan das Nair, Fahim Anwar, Laura Graham, Nima Moghaddam, Robert Crouch, Karen Hoffman, Steve Fallon, Trevor Jones, Isobel Andrews, Ali Gibson, Stephen Timmons

**Affiliations:** 1 University of Nottingham, Nottingham, UK; 2 University of Nottingham Faculty of Medicine and Health Sciences, Nottingham, UK; 3 Centre for Rehabilitation and Ageing, University of Nottingham, Nottingham, UK; 4 Division of Primary Care, School of Medicine, University of Nottingham, Nottingham, UK; 5 SINTEF Building and Infrastructure, Oslo, Norway; 6 Clinical Neurosciences, University of Cambridge, Cambridge, UK; 7 Northumberland Tyne and Wear NHS Trust, Newcastle upon Tyne, UK; 8 University of Lincoln, Lincoln, UK; 9 Health Sciences, University of Southampton, Southampton, UK; 10 Barts and The London NHS Trust, London, UK; 11 Centre for Academic Primary Care, Lifespan and Population Health, University of Nottingham, Nottingham, UK; 12 Centre for Academic Primary Care, University of Nottingham School of Medicine, Nottingham, UK

**Keywords:** REHABILITATION MEDICINE, Implementation Science, QUALITATIVE RESEARCH

## Abstract

**Objectives:**

To explore barriers and facilitators to implementing the ROWTATE vocational rehabilitation intervention and identify lessons for future service delivery.

**Design:**

Qualitative interview implementation study embedded within the development and evaluation of a complex health intervention, guided by the UK Medical Research Council framework.

**Setting:**

National Health Service (NHS) major trauma services in England. The ROWTATE intervention included early vocational assessment lasting up to 12 months, case management, employer engagement and coordinated multidisciplinary support between occupational therapists and clinical psychologists. The intervention was delivered via hybrid models following COVID-19 adaptations.

**Participants:**

Purposively sampled stakeholders including patients, therapists, mentors, employers, carers, commissioners and primary care practitioners.

**Methods:**

80 semistructured interviews were conducted remotely between March 2023 and June 2025 using topic guides informed by the Consolidated Framework for Implementation Research and the Theoretical Framework of Acceptability. Data were analysed using framework coding followed by reflexive thematic analysis to generate cross-cutting themes and implementation-focused recommendations.

**Results:**

Implementation was shaped by six cross-cutting themes that acted as both barriers and facilitators depending on context. Stakeholders highlighted the importance of addressing diverse patient needs and outcomes through early, personalised support and case management. Remote delivery improved accessibility and flexibility but was most effective when combined with face-to-face contact to strengthen relationships and contextual understanding. Multidisciplinary occupational therapy and clinical psychology collaboration enabled holistic care, though required structured processes to overcome communication challenges. Mentoring provided essential professional development and mitigated isolation in remote roles, while employer engagement facilitated workplace adjustments and retention but was constrained by patients’ reluctance to involve employers and by variable occupational health provision. Strategic buy-in from commissioners and clinical leaders was critical, with emphasis on robust outcome data and economic evidence to support sustainability.

**Conclusions:**

Findings inform strategies for embedding vocational rehabilitation within integrated care models and identify core components that should be protected to support implementation when scaling hybrid delivery (eg, mentoring and structured multidisciplinary team processes). The study also outlines scalable approaches to hybrid service delivery, workforce development and employer engagement, offering transferable lessons for health systems seeking to reduce economic inactivity and address post-trauma inequalities.

**Trial registration number:**

ISRCTN43115471.

**STRENGTHS AND LIMITATIONS OF THIS STUDY**

- Inclusion of a wide range of stakeholder perspectives across multiple settings provided a rich and nuanced understanding of factors influencing implementation.
- Methodological rigour was enhanced through double coding and iterative team discussions, strengthening theme development and validation.
- Ongoing patient and public involvement ensured the study remained grounded in real-world priorities and lived experience.
- Recruitment challenges led to underrepresentation of some groups (eg, employers, general practitioners, carers), reducing depth of insight in these areas.
- COVID-19-related adaptations may affect generalisation to non-pandemic settings, though they also reflect a broader shift toward hybrid and digital service delivery.

## Background/introduction

 Injuries in working age adults are common,^1^ and major trauma disproportionately affects individuals of working age, often resulting in physical disabilities,^2^ psychological problems,^3 4^ reduced quality of life^5 6^ and difficulty returning to work^7 8^ including poorer productivity after returning.^9^ In the UK, absence rates from work due to illness or injury have increased, with millions of working days lost annually.^10^ Major trauma is defined as an ‘injury or combination of injuries that are life-threatening and could be life-changing because it may result in long-term disability’.^11^ Major trauma centres (MTCs) save lives but vocational rehabilitation (VR) for trauma survivors is often lacking.^7 12 13^ While UK-specific return to work (RTW) rates vary by injury type and severity, one study showed that only around 66% of individuals had returned to work within 1–3 years postinjury, after moderate to severe injury.^14^ This is supported by a multicentre UK cohort study which found that psychological morbidity, longer hospital stay and threatening life events significantly reduced the likelihood of returning to work between 2 and 12 months postinjury.^15^ A recent systematic review suggests that VR interventions delivered by occupational therapists (OTs) significantly improve RTW outcomes compared with usual care. However, it was not clear which elements were effective in facilitating RTW.^16^

VR is ‘a multiprofessional approach that is provided to individuals of working age with health-related impairments, limitations or restrictions with work functioning and whose primary aim is to optimise work participation’.^17^ Reviews of VR in trauma populations focus mainly on brain or spinal cord injury, much of the evidence is of low quality and only a small number of randomised controlled trials (RCTs) found improved rates of, or time to, RTW.^7 18–21^ Evidence on the effectiveness of VR in patients with a wide range of traumatic injuries exists but is limited in scope.^22–24^ One review^7^ reported 11 VR interventions for those with traumatic injuries and summarised that where VR has been shown to be effective, interventions included individual tailoring, case coordination, setting vocational goals and engaging with employers. However, the review also highlighted a significant gap in robust evaluation and practical guidance for implementation. While it is important to develop effective VR interventions, implementation is critical. Without structured implementation strategies, interventions may never be translated into routine practice or policy.^25 26^ This implementation gap undermines the potential benefits for individuals recovering from trauma and for the wider economy.

Over the past decade, UK policy has increasingly emphasised the integration of health and employment support to address rising economic inactivity linked to ill health. The Work and Health Programme^27^ was launched within the last decade and represented a shift towards personalised employment support for people with disabilities and long-term health conditions. The NHS Long Term Plan^28^ reinforced the role of rehabilitation in enabling independence and participation, including VR as a core component of recovery pathways. Building on this, the 10-Year Health Plan (2025) introduced three major priorities for system transformation: a shift from hospital to community-based care, the adoption of digital technologies and a stronger focus on prevention and early intervention.^29^ Recently, government initiatives such as WorkWell^30^ and Connect to Work^31^ have piloted integrated health and employment services, offering tailored coaching, workplace adjustments and access to local health interventions to support sustained employment for those with complex needs. Policy discourse has shifted toward prevention and early intervention, with proposals for a national occupational health service^32^ and tax incentives to expand employer provision of health-at-work support.^33^ These developments reflect the importance of VR in reducing health-related worklessness, improving productivity and promoting workforce resilience. UK evidence demonstrates that VR delivers a strong business case by reducing health-related worklessness, improving productivity and generating societal and economic benefits.^34^

The ROWTATE intervention was developed within this evolving policy landscape and informed by the evidence base outlined above. This work forms part of a research programme (the ROWTATE programme; NIHR, Ref: RP-PG-0617-20001) and the intervention was tested for feasibility^8 35^ and acceptability,^36^ and is being evaluated for effectiveness and cost-effectiveness in an RCT.

The ROWTATE intervention represents a novel approach, underpinned by a biopsychosocial philosophical approach^37^ and embodied through core evidence-based treatments. The intervention was informed by systematic review evidence, stakeholder engagement and co-design processes, with mechanisms mapped in a published logic model.^7^

In summary, the intervention included a ROWTATE-trained OT contacting patients early after their serious injury providing holistic personalised support targeting the impact the injury had on the person and their job. Patients were assessed by OT for psychological need and referred to a clinical psychologist (CP) where necessary. OTs and CPs focused on the patient’s VR needs, helping patients create VR goals and engaging employers to facilitate a sustainable and monitored RTW for up to 12 months from injury.

The objectives of the analyses presented in this paper were to identify the barriers and facilitators to implementing the ROWTATE intervention and generate recommendations for implementation of the intervention in real world settings.

## Methods

### Study design

The ROWTATE intervention was designed in accordance with the UK Medical Research Council (MRC) framework for the development and evaluation of complex health interventions.^38^ This framework emphasises the importance of ongoing review of context and stakeholder engagement and suggests integrating implementation planning from the outset to facilitate successful adoption during and after the trial. It is now recognised as best practice to evaluate both effectiveness and implementation concurrently, particularly for complex interventions, to address the well-documented time lag between demonstrating efficacy and achieving real-world adoption. The framework explicitly recommends integrating implementation considerations throughout development and evaluation phases to maximise impact and scalability.^39^ A qualitative approach was selected to capture diverse stakeholder perspectives on barriers and facilitators to implementation, generating insights to inform future service design and scalability. Semistructured interviews were conducted with purposively sampled stakeholders. The Consolidated Framework for Implementation Research (CFIR) was used to guide data collection and analysis.^40^

### Setting and context

The study took place in England during implementation of the ROWTATE intervention in a randomised clinical trial. In summary, the intervention included a ROWTATE-trained OT contacting patients early after their serious injury and providing holistic, personalised support targeting the impact of injury on work. OTs assessed patients’ psychological needs as part of a structured holistic assessment of emotional well-being (eg, mood, adjustment, anxiety and trauma-related responses) and referred to a CP where appropriate. OTs and CPs worked together to address VR needs, supporting patients to set goals and engage with employers to facilitate a sustainable and monitored RTW for up to 12 months following injury. Access to CP input varied across sites in line with NHS service provision, which may have influenced participant experiences and is considered in the interpretation of findings.

Delivery of the intervention coincided with the COVID-19 pandemic, which required a change from the originally planned in-person model to an online format during the pandemic and subsequently to a hybrid model. There were also difficulties in recruiting and retaining clinical staff. Initially, the intervention was intended to be delivered across eight NHS sites by locally based staff working within multidisciplinary teams (MDTs). In response to staffing constraints, facilitated by the opportunity of online delivery, a ‘floating’ staffing model was adopted, where clinical staff were not physically co-located with patients and did not routinely work on site with other colleagues. These adaptations significantly altered the planned implementation of the intervention but also generated transferable learning about the feasibility of remote and hybrid rehabilitation models. This learning has potential to inform future service design and workforce planning, contributing to improved resilience and scalability of VR services in the NHS and beyond.

### Public and patient involvement

A patient and public involvement (PPI) group was established at the commencement of the study. PPI contributors were engaged throughout the research process. They co-developed the interview schedule, reviewed participant-facing materials, and co-conducted interviews. This involvement enhanced the relevance, accessibility, and equity of the research process, aligning with NIHR standards for meaningful PPI.^7 41^

### Participants and recruitment

Purposive sampling was used to ensure diversity across stakeholder groups, including patients, therapists, mentors, employers, carers, commissioners and primary care practitioners. We planned to recruit approximately 15 patients, 15 therapists (interviewed at two time points), all mentors (n=5) and 8 of each type of other stakeholder (employers, carers, commissioners and primary care practitioners). Sample size was guided by the concept of information power, which considers study aim, sample specificity and quality of dialogue rather than fixed numerical targets.^42^

Patients, therapists and mentors were eligible if they had direct experience with the ROWTATE intervention and could provide informed consent. All patients and therapists participating in the RCT were invited to express interest in participating and received an invitation pack with study information and consent forms. Patients were purposively sampled to represent variation in site, age, gender and injury severity. Therapists were sampled to reflect different roles, genders and geographical locations. Participants were asked to complete a written consent form in advance of the interview where possible. Where written consent was not feasible (eg, due to timing or practical constraints), verbal consent was obtained and recorded at the start of the interview.

Patients were asked to facilitate contact with their employers, general practitioners (GPs) and carers (eg, family members) to request them to participate in the study. However, consent for employer involvement was limited, and responses from employers with direct experience of the intervention were low. Similarly, GP recruitment was poor, and only one patient consented, and this individual’s carer was subsequently invited and participated. To enable these voices to be included, we expanded the inclusion criteria to include (1) employers with experience supporting individuals with RTW after illness or injury (recruited via the research team’s employer network using email invitations) and (2) clinicians experienced in supporting RTW after illness or injury (recruited via local clinical networks and a university Medical School) to provide a wider system-level perspective on implementation and contextual influences beyond direct intervention exposure.

Commissioners were eligible if they worked in areas served by MTCs involved in the trial and had responsibility for commissioning UK health services. Recruitment was via the research team’s commissioner network using email invitations.

### Data collection

Interview schedules were informed by findings from an earlier feasibility study^35^ the Theoretical Framework of Acceptability (TFA)^43^ and the CFIR.^40^ Questions explored participants’ experiences of the intervention across all domains of both frameworks. The use of CFIR and TFA ensured systematic exploration of factors influencing implementation and acceptability, generating actionable insights for service design, workforce planning and policy development. The interview guides were co-produced by the research team and PPI and copies are available in the online supplemental materials.

Participants who expressed interest in the study received an information sheet and consent form. They were contacted by a researcher to book an interview and provided written or verbal consent prior to the interview. Interviews were conducted via telephone or online platforms, according to participant preference.

Semistructured interviews were conducted between March 2023 and June 2025 by four experienced qualitative researchers (CD, RL, CM and BK), all with backgrounds in health services research and implementation science. One trained PPI contributor (SF) co-interviewed some patients alongside an academic researcher, enhancing inclusivity and relevance. All interviews were audio-recorded and transcribed verbatim using either a University transcription service or automated transcription software. Transcripts were checked, corrected, anonymised and securely stored by the research team in compliance with GDPR and institutional data governance policies.

### Data analysis

Interview transcripts were imported into NVivo V.15 for data management and coding. A hybrid deductive–inductive approach was taken, combining framework-based coding with reflexive thematic analysis, to enable both theoretically informed and data-driven insights.

Analysis followed a two-stage process. Stage 1 involved framework coding. All transcripts were initially coded to the TFA^43^ and CFIR domains^40^ by one researcher (CM), reflecting a deductive approach applied from the outset to organise the data in relation to implementation constructs. To enhance rigour, a 10% sample was double-coded by two additional researchers (BK and RL), with discrepancies resolved through discussion. This process supported dependability and transparency.

Data that did not clearly align with existing TFA or CFIR constructs were coded inductively and retained for further analysis, ensuring that participants’ perspectives were not constrained by the framework.

At stage 2, reflexive thematic analysis following Braun and Clarke’s reflexive approach^44^ was then applied to the framework-coded dataset within and across CFIR domains to identify patterns of barriers and facilitators. This stage involved a more inductive and interpretive analysis, allowing themes to be generated from participants’ accounts while drawing on, but not limited to, the organising frameworks.

Themes were refined through six iterative team meetings involving a multidisciplinary group (KR, DK, ST, JH, RL and BK), incorporating diverse perspectives and reflexive discussion of researcher assumptions. The early and final themes were confirmed through discussion in two meetings with the PPI team (ST, AG, IA, TJ and others). This collaborative approach strengthened confirmability and reduced interpretive bias. Reflexivity was supported through ongoing analytic discussions within the research team, with attention to how disciplinary perspectives and prior assumptions shaped interpretation.

Reporting follows the Consolidated criteria for Reporting Qualitative research (COREQ) checklist for qualitative research.^45^ The completed checklist is provided in online supplemental materials.

## Results

We conducted 80 interviews. Participant interviews, their roles and the characteristics of their interviews are shown by group in table 1.

**Table 1:** Participant summary

Participant ID	Participant role	Type of interview	Total number of interviews
PA01-PA15	Patient	Acceptability	15
PI01-4, PI07-PI15	Patient	Implementation	13
TA01-TA15	Therapist	Acceptability	15
TI01, TI03-TI04, TI06-TI15	Therapist	Implementation	13
ME01-ME04	Mentors	Combined	4
EMP01-EMP06	Employers	Combined	6
COM01-COM07	Commissioners	Implementation	7
CAR01	Carer	Implementation	1
GP01-GP06	Primary care practitioner	Implementation	6
		Total	80

Participant characteristics are shown in tables 2–4.

**Table 2:** Patient stakeholder characteristics

**Patient characteristics (n=15)**	
Gender	Male: 9 (60%) Female: 6 (40%)
Age	Range: 22–71 (Mean: 52.36, Median: 58.5)
Marital status	Single: 5 (33%) Married/cohabiting: 8 (53%) Long-term relationship: 1 (7%) Separated: 1 (7%)
Years of education	Range: 11–20 (Mean: 14.43, Median: 14.00)
Injury	Fractures: 13 (86%) Head injury: 1 (7%) Nerve: 1 (7%) Other: 9 (64%)
Injury severity	9–15: 12 (80%) >15: 3 (20%)
Injury type	Fall: 7 (47%) Road Traffic Accident (RTA): 7 (47%) Other: 1 (7%)
Injury location	Home: 5 (33%) Road: 8 (53%) Work: 0 Educational establishment: 0 Other (boat, street): 2 (14%)
Employment	Employed: 11 (73%) Self-employed: 4 (27%)
Time from injury to interview	Range: 4–20 months (Mean: 13.43 months, Median: 15 months)
Involvement	CP involvement–5 (33%) Employer involvement–7 (47%)
Returned to work	13 (87%)

CP, clinical psychologist.

**Table 3:** Therapist stakeholder characteristics

**Therapist table (n=15)**	
Gender	Male: 2 (13%) Female: 13 (87%)
OT/CP	OT: 12 (80%) CP: 3 (20%)

CP, clinical psychologist; OT, occupational therapist.

**Table 4:** Employer stakeholder characteristics

**Employer (n=6)**	
Gender	Male: 2 (13%) Female: 13 (87%)
Ethnicity	White British (100%)
Size of business (S<50, M=50–250, L=350+ employees)	Small: 1 (17%) Medium: 2 (33%) Large: 3 (50%)
Type of business	Freight: 1 (17%) Banking: 1 (17%) Financial services: 1 (17%) Electrical: 1 (17%) Rail: 1 (17%) Consultancy: 1 (17%)
Employer of ROWTATE participant	Yes: 3 (50%) No: 3 (50%)

Analysis identified six cross-cutting themes that influenced implementation across CFIR domains. Each theme acted as both a facilitator and a barrier depending on context. Illustrative examples for each theme are presented below, highlighting stakeholder perspectives and implications for future service design and policy. A large supplementary data table is offered in supplementary data representing cross-cutting data in each theme and subtheme (see online supplemental table S1).

### Theme 1: patient needs and outcomes

Early support following injury was consistently described by patients as critical to recovery. Stakeholders agreed that early intervention, clear structure and goal-setting were essential for VR. Patients emphasised the need to understand their recovery trajectory, the impact of their injuries and why pacing and phased RTW were important. The ROWTATE intervention was widely perceived as addressing these needs by providing a structured pathway that helped prevent premature RTW or prolonged absence.

We can have a genuine conversation where I really want to learn about what does your day to day look like and what’s daunting about going back into that? And what’s come out from your experiences of the injury and since the injury that we might need to work through or might be a bit of a barrier to you going back to what you were doing before? (TA11)

Patients valued relational and emotional support alongside practical guidance. Many described therapists as a source of motivation and accountability, particularly when family or informal networks were limited. Several patients suggested that they were more receptive to advice from clinicians than from relatives, highlighting the importance of professional input to those without additional support. Those with strong family support also reported that they found clinical involvement helpful, especially in sustaining engagement and motivation. Carers also benefited indirectly, reporting that the ROWTATE intervention helped them support patients more effectively.

She would have carried on and she would have gone [back to work] too soon and then she would have… she could have caused harm to either herself or someone else (CAR01)

Psychological support was identified as a recurring need across many participants. Some participants did not meet thresholds for formal referral to the ROWTATE CPs but still required emotional support, which was provided by OTs. Those referred to the ROWTATE CPs received a multidisciplinary approach, involving both the OT and CP. This was seen as a strength of the ROWTATE intervention, bridging a gap in usual care and embedding mental well-being within VR.

Access to someone like a clinical psychologist is incredibly difficult these days through primary care. So, I guess in the kind of instances from an injury where that is required, again having that input easily and readily available, I think would be almost something of a luxury, but potentially really helpful in terms of you know supporting individuals getting back to work. (GP01)

Flexibility and personalisation were central to the ROWTATE intervention’s success. Patients appreciated responsive care tailored to evolving needs, and therapists developed skills to adapt delivery accordingly. However, therapists noted that complex injuries such as traumatic brain injury (TBI) required more time to provide effective support. Case management was a critical function of the OT role. Patients frequently needed help navigating NHS systems, clinical services and social or financial support. Therapists acted as coordinators, reducing the burden on patients and improving continuity of care, though this role was challenging given the complexity of service landscapes.

There’s such a strong emphasis on advocating for people once they’re at home, and I learnt so much about what life is like after hospital and how we can continue to support them. (TA13)

Patients, therapists and employers agreed that support for workplace communication and adjustments was often lacking in usual care. The ROWTATE intervention helped bridge this gap, particularly where occupational health provision was limited or inaccessible. Engagement with employers varied and while some patients had highly supportive employers and required minimal intervention, others relied heavily on the ROWTATE intervention to negotiate phased returns to work and workplace adaptations.

[The OT said] let’s sit down and have a conversation about what that could look like, then I can explain it to your employer as well and if he’s got more questions that are more related to your role then we can have a conversation as a group. (PA12)

Patient engagement with the ROWTATE intervention itself was also variable. Therapists reported that non-engagement reduced the time available for delivering intended support. Barriers that reduced engagement included clinical factors (eg, TBI, pain, ongoing surgeries) and contextual challenges such as financial pressures or legal claims that disincentivised RTW. Strategies to improve engagement included flexibility in allowing patients to participate at their own pace and practical measures such as appointment reminders and flexible appointment rescheduling.

Stakeholders consistently highlighted that postdischarge needs for trauma patients are often unmet by existing NHS or social care services. The ROWTATE intervention was seen as addressing this gap, providing continuity and reassurance during a vulnerable period. Participants used strong language to describe the importance of this support, reinforcing its perceived value.

It’s almost as if when you walk out of the hospital the medical staff wash their hands of you and that’s it… The only person I had contact with was [the OT]. She was phoning up, and for me, that was a comfort blanket, that was fantastic. (PA05)

RTW was seen as a priority outcome by commissioners and employers, who linked it to health, cost-effectiveness and wider social benefits. Some patients reported that the ROWTATE intervention facilitated RTW in cases where this might not otherwise have occurred, including transitions into new roles.

Within two weeks of our last phone call I was back in work again…I think I probably would’ve still been off sick. (PA11)

Therapists and mentors acknowledged RTW as a useful proxy for broader benefits such as confidence, independence and well-being. They also reported that fidelity to the ROWTATE intervention design would be essential for sustaining positive outcomes, though resource constraints and commissioning priorities were noted as potential barriers.

In summary, the ROWTATE intervention addressed a wide range of patient needs through early intervention, structured rehabilitation, psychological support and assistance with employer engagement. Its flexibility allowed responsiveness to diverse and evolving needs, filling a recognised gap in usual care. Stakeholders were broadly positive about its impact, though there was variability in patient engagement. Evidence suggests the intervention contributed to successful RTW outcomes and broader improvements in well-being, reinforcing its relevance for future service models and policy.

### Theme 2: remote delivery

The ROWTATE intervention was originally designed for face-to-face delivery but was adapted for remote provision during the COVID-19 pandemic. This unplanned shift became a cross-cutting issue, impacting multiple aspects of implementation. While remote delivery introduced challenges, it was predominantly viewed as a facilitator, improving accessibility and flexibility for both patients and therapists.

Before Covid [online appointments] would’ve been like really alien wouldn’t it, it would’ve been completely brand new. Whereas because of Covid I think … we’ve adapted a little bit more to doing a lot more on Teams meetings and virtual meetings rather than seeing people face-to-face. (TA03)

COVID-19 acted as a catalyst for telehealth adoption. Patients and therapists reported increased confidence in virtual appointments due to changing digital habits during the pandemic. Many patients described remote sessions as convenient and less physically demanding, reducing barriers such as travel, cost and fatigue. For some, remote delivery was preferable, particularly in the early stages of recovery when mobility was limited. Therapists agreed with these benefits, noting that remote delivery enabled greater reach, time efficiency and flexibility for scheduling especially for patients balancing rehabilitation with work commitments.

I think it would’ve been a bit more tiring as well if it was having to go to a physical location, because you would have to get ready, remember you’ve got to be somewhere, you’ve got to plan your journey, you’ve got to plan getting home, so that would be quite overwhelming initially I think. So, but for me it worked really, really, really well. (PA02)

Remote delivery also supported innovative workforce models. Floating therapists, who were not tied to a single site, could deliver care across regions, helping to address supply-demand challenges. This adaptability was seen as a strength of the ROWTATE intervention, allowing continuity of care despite pandemic-related restrictions.

I think you’re able to reach a lot more people. It’s definitely more time efficient, because you’re not having to spend time driving or getting any kind of transport. So definitely able to reach more people and more efficient time. I think it’s been quite useful for my participants as well, because sometimes they’re quite flexible as well, I think with what time sessions might be. Because if they’re at work, like a few of them have really supportive employers, but they can still go to their days of work but might tell their employer that they’re taking their hour lunch and go into an office and have a session with me… if it was in a clinic you might have to block off three or four hours or take a full day off work. (TA13)

While remote care improved access, a hybrid model combining remote and in-person appointments was often preferred. Therapists reported that face-to-face visits provided richer contextual understanding of patients’ home and work environments and strengthened therapeutic relationships. Remote delivery sometimes reduced non-verbal cues and made it harder to assess issues such as cognitive fatigue. Some therapists also noted higher rates of missed appointments online and expressed concern that remote delivery had become the default mode without sufficient consideration of patient preference or clinical need.

I think it’s a lot easier to cancel someone who you’ve never met, who you just speak to over a screen as opposed to cancelling someone who turns up at your house …every two or three weeks and you have that bit of a rapport with. Like a couple of mine just haven’t turned up and haven’t told you in advance that they’re going to be there. (TA03)

Remote working affected team dynamics as well. Facilitating collaboration was more challenging when staff were remotely located, and therapists working outside local NHS Trust teams found it harder to navigate systems and build relationships. Mentoring and structured communication were identified as important mitigators of these challenges.

My challenge has been that I’m a floating OT, so I’m not in one area. So, I have clients [in 4 different geographic areas], and finding information out, or who they’re also involved with, can be quite challenging for me because, geographically, you don’t know it. But actually, because the role is virtual and there’s email, that’s not tricky once you get the information, and the onsite OTs are really helpful at providing that information. (TI08)

In summary, remote delivery of the ROWTATE intervention was widely perceived as a facilitator of implementation, enhancing accessibility for patients and flexibility for therapists. It enabled broader reach and more efficient use of resources, supporting continuity during the pandemic and beyond. However, challenges related to communication, contextual understanding, and team integration highlighted the importance of a hybrid model and ongoing support structures such as mentoring. These findings have implications for future service design, particularly in scaling digital and hybrid rehabilitation models within the NHS.

### Theme 3: multidisciplinary support

The integration of OTs and CPs working as a MDT was a distinctive feature of the ROWTATE intervention and widely regarded as a key facilitator of successful implementation. This collaborative model enabled holistic support, addressing both physical and psychological rehabilitation needs and this was an approach valued by patients, therapists, employers, primary care practitioners, and commissioners.

I’m heartened by the option of referring into a psychologist because I do think with my previous experience … people following major trauma once they had been through the physical health services… it was often after rehab that the psychological impact would hit people, it would then be left to get so bad that they ended up coming to secondary mental health services … So, I think the psychological aspect is really important. (COM07)

Patients described the benefits of having a coordinated team that could respond to complex needs, while therapists and mentors highlighted that joint working created a more comprehensive and patient-centred pathway. Best practice examples demonstrated that strong communication between OTs and CPs enhanced continuity of care and problem-solving, reinforcing the intervention’s goal of supporting RTW.

I got the enhanced support, so I got support from the clinical psychologist as well… my OT and CP would meet separately to discuss and catch-up and then my OT, my employer and I would have a three-way Teams’ call. (PA12)

However, working as an MDT required time, effort, and intentional collaboration. Where OTs and CPs were co-located, relationships developed more naturally, facilitating communication and shared understanding. In contrast, remote and ‘floating’ delivery models introduced challenges, including scheduling difficulties, reduced informal contact, and unfamiliarity with local systems. These barriers sometimes led to missed opportunities for joint input or delays in care.

In [one area] because the clinicians are working very loose hours, or very restricted hours, I think it’s difficult for them to communicate amongst each other. So there have been times that I’ve picked up and somebody else has said they were going to pick up, and then it’s all got very messy. (TA10)

Role clarity was a barrier on some occasions. While CPs were accustomed to managing their own caseloads, some OTs needed encouragement to take the lead in coordinating VR. Mentoring emerged as an important enabler, providing a virtual space for peer support and helping therapists, especially those working in portfolio or remote roles, to develop confidence in MDT working. This is explored further in theme 4 (mentoring).

Participants emphasised that fidelity to the intervention design was critical for achieving positive outcomes. MDT collaboration was seen as central to delivering holistic care and accelerating recovery, but resource constraints and competing priorities risked undermining this aspect of the model. Commissioners and clinicians agreed that sustaining MDT working would require organisational commitment and investment in workforce capacity.

I think we, you know, we see this with reablement, you know, reablement delivery in England. Actually it’s very difficult to get any evidence that it works because actually most of it’s delivered by private companies you’re using, so support workers. And not overseen or led by occupational therapists but where we see that we do see positive impacts. So you know if you give them too free a hand then they’ll come up with the cheapest, you know, delivery possible and it’s not going to do the job. (COM07)

In summary, the MDT approach was a novel and valued component of the ROWTATE intervention, enabling integrated physical and psychological support for patients. While strong examples of collaboration were evident, challenges such as communication barriers, scheduling and role clarity, particularly in remote contexts, highlight the need for structured processes and ongoing support. Mentoring and organisational buy-in were identified as key strategies for embedding MDT working into routine practice.

### Theme 4: mentoring

Mentoring was a core component of the ROWTATE intervention and widely regarded by therapists as essential for successful implementation. Mentors brought extensive experience in VR, research and supervision, providing therapists with structured support, professional development, and reassurance particularly during the early stages of the therapist role. This helped build confidence, develop skills, and foster a sense of belonging within the team.

I think just helpful to have someone to talk to if you’re not sure about something. And, you know, especially people with experience, because I know (Mentor name) has got a lot of experience… We’ve got enough support because you’ve got quite a good structure as well that you can follow. (TA02)

Mentoring was delivered online through scheduled individual sessions and peer groups, creating opportunities for shared learning and reflective practice, as well as ad hoc personalised support for therapists as required. Therapists valued group sessions for problem-solving, knowledge exchange, and the chance to reflect on clinical decisions.

The thing that they find most helpful about mentoring is this space and time for reflective practice, so that they can really think, ‘What am I doing with this person? Am I doing it in the best way? What improvements could I make? How could I change what I’m doing to get the best outcome?’ Whereas, normally in their everyday clinical, they’re in the acute sector mostly, it’s just the same thing all the time…and there’s no time for them to do any reflective practice. (ME02)

For therapists working remotely or in floating roles, mentoring created a virtual team environment that mitigated isolation and strengthened cohesion.

I see us as being all one big department… I get to know all of them… so it’s all nice really, it all feels like a big—I won’t say happy family, but it feels like a nice big team, rehab team. (ME02)

Despite these benefits, some barriers were identified. Engagement varied, potentially because mentoring was not mandatory or monitored, and mentors sometimes needed to follow-up with therapists to maintain participation. Scheduling was challenging due to unpredictable workloads and portfolio working, increasing demand for flexible provision. Professional differences between OTs and CPs in supervision and CPD expectations occasionally created misalignment, requiring clarity on roles and expectations.

In summary, mentoring was a foundational element of the ROWTATE intervention, supporting therapist confidence, skill development, and team cohesion. It enabled reflective practice, knowledge sharing, and virtual team-building—particularly for those in remote roles. While challenges related to engagement, scheduling and professional differences were noted, mentoring was widely regarded as essential to successful implementation and should be considered a core component of future VR models.

### Theme 5: employer engagement

Employers played a pivotal role in the success of the ROWTATE intervention. They described the challenge of balancing organisational priorities with the well-being of employees recovering from major trauma, describing a dual responsibility often complicated by uncertainty around recovery timelines and workplace adjustments. This uncertainty was identified as a major barrier to effective planning. The ROWTATE intervention was widely regarded as a valuable resource for navigating these complexities, supporting informed decision-making that benefited both the organisation and the individual.

You’d want to know enough information to make the right decisions for the good and benefit of that individual and the company as well. You’ve got to protect the company’s interest as well in this. (EMP03)

The ROWTATE intervention demonstrated adaptability across diverse employment contexts, from desk-based roles to physically demanding jobs in sectors such as healthcare, manufacturing and transport. Employers and therapists agreed that flexibility was a key strength enabling tailored support for RTW planning in a wide range of contexts.

What you find is that very small organisations were good, because they’re [based on] human relationships, and [staff are] mutually supportive one to another because they all tend to be good friends. And very big organisations tended to be good, because they had seniors and professional people to implement and check those procedures. But the majority of people are in between, and they neither have the human empathies and support or the procedures in place. (EMP02)

While some suggested that company size and culture influenced implementation, others felt that the ROWTATE intervention could fit in organisations of any size. Larger employers with established well-being policies or occupational health services saw the ROWTATE intervention as complementary, while smaller organisations valued the external expertise it provided. Several employers noted its potential as a private offering for businesses seeking enhanced support for staff.

Employers consistently reported that, despite strong motivation to support employees, they often lacked the knowledge or tools to do so effectively. The ROWTATE intervention helped bridge this gap by clarifying medical considerations, advising on reasonable adjustments, and facilitating communication between employees and managers. This was particularly valued in safety-critical roles, where confirmation of fitness to work was essential.

Potentially involving the employer I think is really helpful and really valuable in terms of obviously hopefully having their input from an early stage as well because obviously there’s going to be a huge amount of individualisation in terms of, I guess you know what the injury is, what role an individual does and how the two sort of things link in terms of what they can or can’t do going forwards. (GP01)

Clinicians, commissioners and employers highlighted alignment with current policy drivers to reduce economic inactivity, suggesting that national initiatives could further incentivise uptake.

Barriers to employer engagement included patient reluctance to involve their workplace, concerns about employer attitudes, and variability in occupational health provision. Some participants preferred therapists to communicate indirectly through them, while others welcomed direct engagement. Where occupational health services existed, their quality and relevance varied but most stakeholders perceived the ROWTATE intervention as offering more specialised and practical support than traditional occupational health, particularly for trauma-related needs.

To be honest, I’m not a huge fan of occupational health services from this job or in previous roles, because it’s like you give them the watch and they tell you the time. (EMP01)

In summary, employer engagement was a key component of the ROWTATE intervention, enabling workplace adjustments and supporting organisational planning. The ROWTATE intervention helped employers manage uncertainty, improve communication and retain staff, offering a valuable complement—or alternative—to existing occupational health provision. While engagement varied and was sometimes limited by participant preference or organisational capacity, the adaptability of the ROWTATE intervention across roles and sectors was a major strength. Government and organisational policies were identified as important facilitators for scaling and sustaining employer engagement in future VR models.

### Theme 6: stakeholder buy-in and strategic alignment

Achieving successful implementation of the ROWTATE intervention will require commitment from key stakeholders, particularly commissioners and clinical leaders. Commissioners consistently identified RTW as a priority outcome, linking it to health, social care and economic productivity. Although rehabilitation was not always viewed as a commissioning priority, RTW was seen as a cross-cutting issue with clear system benefits. The holistic nature of the ROWTATE intervention was perceived as compatible with the aims of Integrated Care Boards (ICBs), particularly around integrated, preventative care and improving patient flow from hospital to home. Commissioners emphasised that economic considerations drive decision-making and assigning financial value to broader benefits was suggested as critical for strengthening the business case.

What’s the impact of someone who actually we can help support get back into a job, and them actually not require long term care at home, which the local authority would in the ultimately be funding there. And so, it’s kind of that cost benefit (COM01)

Stakeholders highlighted the importance of robust outcome data and digital tools to support planning and demonstrate impact. Commissioners recommended that the ROWTATE intervention collect and report data on RTW and wider outcomes to facilitate alignment with local priorities. Technology-enabled monitoring was seen as a facilitator for scaling and sustaining the intervention.

I think [you need] a really clear understanding of what data needs to be collected, what measures need to be collected and understanding the electronic systems that are working within. (COM07)

Barriers to buy-in included financial pressures, competing priorities and structural instability within commissioning systems. Commissioners described the challenge of keeping rehabilitation on the agenda in a context of constrained budgets and organisational change. High turnover within ICBs and uncertainty about future structures made relationship-building difficult.

I would say is the fact that basically there’s so many competing priorities within NHS England and or ICBs, it’s the biggest challenge is keeping rehab on that agenda at all. (COM04)

Clinical leaders in NHS Trusts and primary care were identified as potential advocates, but engagement varied. Clinicians noted that time and resource constraints could limit their involvement, though they recognised the ROWTATE intervention’s potential to reduce long-term burden on health and associated costs.

I guess in GP land we have two currencies. One is money and the other one is time and time is the biggie, you know. (GP04)

Therapists and commissioners agreed that generating support from local NHS Trusts required time, clarity of roles and sustained effort. Barriers included role overlap, inertia and negative attitudes within some organisations. Stakeholders stressed that successful implementation of the ROWTATE intervention would depend on strong relationships, clear communication and alignment with local needs along with policy planning.

So, it’s all about all about community services, integrated care, improving flow and then also how we developed joined up community services. So that kind of integrated holistic, more proactive preventative care (COM03)

In summary, stakeholder buy-in emerged as a critical determinant of implementation of the ROWTATE intervention. Commissioners and GPs valued the ROWTATE intervention’s focus on RTW and its compatibility with integrated care priorities but emphasised the need for a strong economic case supported by robust outcome data. Financial pressures, structural instability and competing priorities present significant barriers, underscoring the importance of strategic engagement with commissioners and clinical leaders. Aligning the intervention with policy drivers and demonstrating system-wide benefits will be essential for scaling and sustainability.

## Discussion

### Summary of findings

This study explored the implementation and acceptability of the ROWTATE VR intervention for adults in England recovering from major trauma. Findings indicate that the ROWTATE intervention addressed a critical gap in usual care by providing early, personalised support, psychological input and employer engagement. Implementation of the ROWTATE intervention was or would be shaped in the future by contextual factors, including the COVID-19 pandemic, workforce constraints and commissioning priorities. Cross-cutting themes included patient needs and outcomes, remote delivery, multidisciplinary support, mentoring, employer engagement and stakeholder buy-in. Key factors influencing implementation of the ROWTATE intervention were operational (eg, joint working, mentoring, remote delivery) and outcome-related (eg, participant experience, engagement and RTW outcomes).

### Comparison with existing literature

Our findings reinforce evidence that early, structured VR improves RTW and well-being outcomes following traumatic injury.^7 17^ The emphasis on holistic, biopsychosocial care aligns with recommendations from the UK National Institute for Clinical Excellence major trauma guidelines and international reviews highlighting the need for integrated physical and psychological rehabilitation.^15^ However, VR remains underdeveloped in UK trauma pathways, with provision often fragmented or absent.^13^

Multidisciplinary working and mentoring were critical enablers of implementation of the ROWTATE intervention. MDT collaboration between OTs and CPs supported holistic care, while mentoring enhanced therapist confidence and fidelity. These findings echo previous research on complex interventions, which emphasises structured support and organisational commitment as prerequisites for sustainability.^39^ Depending on the availability of mentoring expertise, future implementation may require organisations to buy-in mentoring expertise from an external provider to embed learning within the organisation and ensure fidelity.

Although the ROWTATE intervention was originally designed for in-person delivery, remote provision became a key facilitator, driven by participant acceptance and convenience. This reflects broader trends in UK healthcare, where telehealth interventions have been successfully implemented post-COVID, supported by increased participant openness to digital care models.^46^ Remote provision was widely perceived as improving accessibility and efficiency, consistent with emerging evidence on tele-rehabilitation.^22 46 47^ However, challenges related to therapeutic relationships and contextual assessment underscore the importance of hybrid models that combine remote and in-person care.

Outcomes are a critical driver of implementation success, yet selecting the most appropriate measure remains challenging. While RTW offers clear economic value, it may not fully capture broader impacts such as quality/purpose of life or long-term well-being. Future service delivery models and evaluations should integrate functional and psychosocial measures to broaden assessment of intervention impact.

### Interpretation and implications

Securing commissioner or funder buy-in for interventions like the ROWTATE intervention requires alignment between demonstrable outcomes, stakeholder engagement and commissioning priorities. The emphasis on RTW aligns with NHS and government strategies that recognise its economic and social value. NHS England’s Long-Term Plan^28^ and Fit for the Future: 10-Year Health Plan^29^ set out three transformative shifts: from hospital to community, from analogue to digital and from sickness to prevention. The ROWTATE intervention operationalises these priorities by delivering community-based, digitally enabled rehabilitation and addressing work-related health needs early to prevent unemployment-related ill health and other adverse outcomes. Recent initiatives such as WorkWell^30^ and Connect to Work^31^ further underscore the policy imperative for integrated health and employment support. However, these initiatives provide much less intensive support than the ROWTATE intervention and are unlikely to meet the complex VR needs of many patients after traumatic injury.

Outcomes drive implementation success, but selecting the most appropriate outcome measure is challenging. While our study focused on RTW as our primary outcome, this may not fully capture broader impacts such as psychological well-being, quality of life or purpose in life which we included as secondary outcome measures.^48^ Literature suggests that although RTW outcomes offer clear economic value, they may not reflect the full scope of patient recovery. Future evaluations should consider integrating both functional and psychosocial measures to better assess intervention impact.^49 50^

Commissioners emphasised the need for robust outcome data and digital tools to support planning and demonstrate impact. Technology-enabled monitoring and employer communication platforms could facilitate scaling and sustainability. Securing commissioner or funder buy-in for interventions like the ROWTATE intervention requires alignment between outcomes, stakeholder engagement and public commissioning priorities. NHS England’s rehabilitation guidance highlights the importance of outcome-driven, transferable models that can be adapted across settings. The identification of the ROWTATE intervention’s components that should be prioritised offers a scalable approach that supports broader implementation across the NHS.^49–51^

#### Recommendations for future implementation

Our findings highlight several components of the ROWTATE intervention that were most critical for successful implementation, summarised in box 1. Organisations planning to adopt similar interventions should prioritise the elements outlined in the table below:

Box 1 **Recommendations for implementation**

**Practical recommendations for implementation**

**Components to prioritise**

**Early and structured engagement with case management**

- Contact patients soon after discharge; set vocational goals and phased return to work (RTW) plans.
- Support to connect with wider support services, employer, family, etc.

**Employer involvement**

- Initiate communication early; provide patient and employer with the knowledge and practice to support workplace adjustments and a phased return (where possible).

**Mentoring**

- Embed structured mentoring and reflective practice for therapists to maintain fidelity and confidence.

**Multidisciplinary team (MDT) working:**

- Ensure occupational therapists (OTs) and clinical psychologists (CPs) collaborate closely with clear role definitions and regular joint patient reviews.

**Hybrid delivery**

- Offer remote and in-person options based on patient preference and clinical need.

**Data and digital infrastructure**

- Collect robust outcome data (RTW rates, well-being measures) to demonstrate impact.
- Use digital platforms for scheduling, monitoring and employer communication to streamline processes and support scalability.

**Operational strategies**

- Clarify MDT roles (OTs and CPs) and schedule regular joint patient reviews.
- Collect robust outcome data (RTW rates, well-being measures) to demonstrate impact.

**Strategic alignment**

- Map intervention goals to local Integrated Care Board priorities and national initiatives for example National Health Service (NHS) Long Term Plan, Fit for the Future: 10-Year Health Plan, WorkWell and Connect to Work.
- Prepare an economic case emphasising cost savings from improved health and social care outcomes and economic inactivity.

These recommendations reflect the most critical components identified by stakeholders as well as strategies for data collection and policy alignment. Implementation teams should use these as a blueprint for planning and scaling VR services for patients with traumatic injuries.

Our findings reinforce that success depends not only on intervention design but on organisational readiness and structured support. Mentoring and (OT and CP) MDT collaboration emerged as important for fidelity, while digital infrastructure and robust outcome monitoring were highlighted as essential for sustainability. Aligning intervention goals with NHS priorities and national initiatives will strengthen commissioning cases and facilitate adoption. The identification of the ROWTATE intervention’s components that should be prioritised offers a scalable approach that supports broader implementation across the NHS.^49–51^

### Future research

Further research should evaluate the cost-effectiveness and scalability of VR interventions in routine practice, including their impact on health, social and economic outcomes. Studies should explore digital innovations for remote MDT working and employer engagement, as well as strategies for improving patient engagement and equity of access. Future research should also explore barriers to recruiting and engaging carers, whose role in supporting VR is important but remains under-researched. Comparative research across international contexts would enhance understanding of how policy and system factors influence implementation.

### Strengths and limitations

A key strength of this study is the inclusion of a wide range of stakeholder perspectives across multiple settings, offering a rich and nuanced understanding of the factors influencing implementation of VR after traumatic injury. Methodological rigour was strengthened through double coding and iterative discussions within the research team, which helped refine and validate emerging themes. The involvement of PPI contributors throughout the study further ensured that the research remained grounded in the priorities and experiences of those most affected.

One limitation is that the findings are situated within the UK policy and healthcare context and may not be directly transferable to other international settings. Some recruitment challenges meant that some stakeholder groups were underrepresented, particularly employers and GPs with direct experience of the intervention, who often spoke hypothetically rather than from practice. In addition, variation in access to CP services across sites, including differences in availability and waiting times, may have influenced participant experiences of the intervention and should be considered when interpreting the findings. We were also only able to include one carer, limiting the depth of insight from this perspective. In addition, adaptations made during the COVID-19 pandemic may restrict applicability to non-pandemic conditions; however, these changes arguably reflect a broader and ongoing shift towards digital and hybrid models of health service delivery, which may enhance future relevance.

## Conclusions

The ROWTATE trial shows that a multidisciplinary VR intervention was implemented within trial conditions within the NHS, using predominantly remote delivery. We describe a transferable model for embedding VR within NHS trauma pathways and identify core components that should be protected to support fidelity when scaling hybrid delivery.

Our findings inform strategies for embedding VR within integrated care models and demonstrate how early, personalised support could improve recovery and RTW outcomes. The study highlights scalable approaches for hybrid service delivery, workforce development and employer engagement, offering transferable lessons for health systems seeking to reduce economic inactivity and address post-trauma inequalities.

## Supplementary material

10.1136/bmjopen-2026-118198 online supplemental file 1

10.1136/bmjopen-2026-118198 online supplemental file 2

10.1136/bmjopen-2026-118198 online supplemental file 3

## Data Availability

Data are available on reasonable request.
