# Supplementary material for: Implementation barriers and facilitators to a vocational rehabilitation intervention after traumatic injury (ROWTATE) in the UK: qualitative interviews with key stakeholders
Source: BMJ Open. 2026 Jul 30;16(7):e118198. doi: 10.1136/bmjopen-2026-118198 (PMC13422936; doi:10.1136/bmjopen-2026-118198)
Supplement: online supplemental file 1 [file bmjopen-16-7-s001.docx]

**ROWTATE Interview Schedules**

**Contents:**

Contents

[Patient Acceptability 2](#_Toc221632065)

[Therapist Acceptability 4](#_Toc221632066)

[Employers 6](#_Toc221632067)

[Patient Implementation 9](#_Toc221632068)

[Therapist Implementation 10](#_Toc221632069)

[Carer 12](#_Toc221632070)

[GPs and AHPs 13](#_Toc221632071)

[Commissioners 16](#_Toc221632072)

## Patient Acceptability

**Over the past few months you have been receiving help/advice/support from an occupational therapist (and clinical psychologist if relevant) to help you return to work as part of the ROWTATE study. These questions are about the help/advice/support you have received:**

**General:**

- Can you start by telling us about your experience of the help/advice/support you have received as part of ROWTATE?
- Can you tell us about your experience of your employer taking part in ROWTATE?
- Can you tell us about your experience of working with your OT/CP?

Affective attitude

- How did you/do you feel about the help/advice/support you received from the OT (and CP if relevant)?

Burden

- How much of an effort is/was it for you to engage with the help/advice/support you received?

Ethicality

- Was there any help/advice/support that didn’t feel right to you or that you disagreed with?
- Did the help/advice/support have any negative consequences for you?

Intervention coherence

- What help/advice/support have you received from the OT (and CP if relevant)?
- What do you think the help/advice/support is trying to achieve?

Opportunity costs

- What has it cost you in terms of money, time, other resources to engage with the help/advice/support from the OT (and CP if relevant)?

Perceived effectiveness

- How well do you think the help/advice/support is working/has worked for you?

Self-efficacy

- How confident are you that you can act on the help/advice/support from the OT (and CP if relevant)?

Remote delivery of the intervention:

- What do you think about getting the help/advice/support from the OT (and CP if relevant) via phone/videocalls/other remote methods?
- What were the challenges to getting the help/advice/support in this way?
- How do you think getting help/advice/support by phone/videocall etc affected your relationship with your OT/CP?
- Overall, how well did the technology work for you?

If experienced any difficulty with technology:

- - What difficulties did you encounter and how could they be overcome in the future?
- Was there any technology/devices/software that would have helped but didn’t have?
- If you had a choice in future to have help/advice/support provided face-face or by phone/videocalls/other remote methods, which would you prefer and why?
- Is there anything else you’d like to tell us about the help/advice/support you received by phone/videocall/other remote methods

Is there anything else you would like to tell us about your experience of ROWTATE at this point?

Prompts:

- * Tell me more about that …
- * How did that feel?
- * Can you give me an example of …
- * What do you mean by …

## Therapist Acceptability

**General:**

- Tell us about your experience of providing help/advice/support to patients and their employers during ROWTATE
- What did you find worked best?
- What did you find did not work that well?
- If you could make changes to the help/advice/support in ROWTATE, what would you change and why?

Affective attitude

- How do you feel about the help/advice/support you have provided during ROWTATE?

Burden

- How much of an effort was it for you to provide the help/advice/support during ROWTATE?

Ethicality

- To what extent does/did the help/advice/support you provided during ROWTATE feel right to you or fit with the values of your employing organisation?
- Did providing the ROWTATE help/advice/support have any negative consequences for you, your patients or their employers?

Intervention coherence

- What support/help/advice have you provided for patients and employers during ROWTATE?
- What is/was the help/advice/support you provide in ROWTATE trying to achieve?

Opportunity costs

- What has it cost you in terms of money, time, other resources to provide ROWTATE help/advice/support?

Perceived effectiveness

- How well do you think ROWTATE help/advice/support is working/has worked?

Self-efficacy

- How confident are you that you can provide ROWTATE help/advice/support to patients and their employers?

**Remote delivery of the intervention:**

- What do you think about providing the ROWTATE help/advice/support via phone/videocalls/other remote methods?
  - What challenges did find in doing this?
- Are there any groups of patients that you think remote help/advice/support is less suitable for?
  - Why is this?
  - How might difficulties with remote provision with these patients be overcome in the future?
- How do you think providing help/advice/support remotely affected your relationship with your patient?

If any negative impacts mentioned:

- - How do you think this affected how well your patient engaged with the help/advice/support?
- Did you provide any help/advice/support face-face with patients or employers?
  - Why did you choose face-face for this help/advice/support?
- Overall, how well did the technology work for providing help/advice/support remotely?

If experienced any difficulty with technology:

- - What difficulties did you encounter and how could they be overcome in the future?
- How well did the ROWTATE training prepare you to provide help/advice/support for patients and employers remotely?
- If you had a choice in future to provide the ROWTATE help/advice/support face-face or by phone/videocalls/other remote methods, which would you prefer and why?
- Is there anything else you would like to tell us about your experiences of providing the ROWTATE help/advice/support remotely?

## Employers

**About the employing organisation:**

- Tell us about your organisation
  - Size of organisation
  - Type of organisation (public sector, private sector, third sector, other)
  - Nature of the organisation (e.g. education, services, construction)
  - Types of job roles in your organisation
- Does your organisation have a policy to support those returning from long term sickness absence?
  - If yes, what does this include?
- How does your organisation support managers with managing sickness absence?
  - Provides training?
  - HR staff who support managers with managing sickness absence?
- Does your organisation offer company sick pay (above statutory sick pay) for those on long term sickness absence? If yes, for how long?

**About your experience of the ROWTATE programme:**

***Over the last few months your employee (name) has been receiving help/advice/support from an OT (and CP if applicable) to return to work as part of the ROWTATE project. The ROWTATE project also provides help/advice/support for employers to help their employees return to work. The next few questions are about this help/advice/support:***

- Have you received any help/advice/support from an occupational therapist or psychologist to help your employee return to work?

If yes:

- - Who provided this help/advice/support?
  - Tell us about your experience of getting this help/advice/support
  - What help/advice/support worked best?
  - What help/advice/support did not work that well?
- Was there any help/advice/support that you didn’t receive, which would have been helpful?
  - Would it have been helpful if a workplace visit had been done to provide help/support/advice?
  - What kinds of help/advice/support would a workplace visit have been useful for?
- What support was your organisation able to offer the employee to help them return to work?
  - Financial
  - Access to services (occupational health, counselling, physiotherapy etc)
  - Work adjustments
  - Providing equipment or aids
  - Phased returns/part time working
  - Redeployment
  - Changes to sickness absence triggers, other aspects of usual policies and procedures
  - Access to work, transport
  - Coaching, additional training, accessibility software
  - Others
- If you could make changes to the ROWTATE help/advice/support, what would you change and why?

Affective attitude

- How do you feel about the help/advice/support you and your employee were provided with?

Burden

- How much of an effort was it for you, or your organisation to engage with the help/advice/support from the OT (and CP if relevant)?

Ethicality

- To what extent does/did the help/advice/support feel right to you or fit with the values of your organisation?
- Did the help/advice/support have any negative consequences for you, your organisation or your employee?

Intervention coherence

- What help/advice/support did you receive from the OT (and CP if relevant)?
- Were any workplace visits made by the OT (and CP if relevant)?
- What do you think the help/advice/support is trying to achieve?

Opportunity costs

- What has it cost you, or your organisation in terms of money, time, other resources to engage with the help/advice/support from the OT (and CP if relevant)?

Perceived effectiveness

- How well do you think the help/advice/support is working/has worked?

Self-efficacy

- How confident are you that you or your organisation can act on the help/advice/support you have been given?

**Remote delivery of the intervention:**

- Assuming most of your contacts with the OT (and CP if relevant) were by phone/videocall/other remote methods, how well did that work?
- How well did the interactions with the OT (and CP if relevant) work?
- Overall, how well did the technology work for getting help/advice/support remotely?

If experienced any difficulty with technology:

- What difficulties did you encounter and how could they be overcome in the future?
- If you had a choice in future to have help/advice/support face-face or by phone/videocalls/other remote methods, which would you prefer and why?
- Is there anything else you would like to tell us about your experiences of getting remote help/advice/support for your employee to return to work?

## Patient Implementation

How have things been since we last spoke?

*Relative Advantage*

- What usual care did you receive?
- How do you think ROWTATE compares to the usual care you have received in the NHS? Why?

*Complexity*

- How does ROWTATE compare to usual care with regard to ease of use/delivery?

*Patient Needs and Resources*

- What sort of injuries did you have?
- What sort of rehab activities did you do?
- How well did ROWTATE meet your needs?
- Are there any barriers to meeting your needs? What helped?

*Culture*

- What sort of company did you work for?
- How would you describe the culture of your employer/employing organisation?
- What sort of support did your employer offer you?
- How was / is the support from your line manager?
- Was your employer involved with ROWTATE? Why or why not?

*Implementation Climate*

- Do you think your employer and line manager was in a good place to implement ROWTATE?
- Why do you think that?
- Was your organisation prepare to help someone return to work?
- Do you think ROWTATE was compatible with your employing organisation and their mission?

*Knowledge and Beliefs About the Intervention / Self-Efficacy / Individual Stage of Change/Other Personal Attributes*

- What do you think overall about ROWTATE?
- How well does ROWTATE work, in your experience?
- How confident are you that you can carry out the actions recommended by the Occupational Therapist/Clinical Psychologist
- Is there anything else you would like to tell us about your experience of ROWTATE?

*Can we contact your GP, carer and employer for interview?*

## Therapist Implementation

How have things been since we last spoke?

*Relative Advantage*

How do you think ROWTATE compares to usual care? Why?

*Adaptability*

How do you think ROWTATE has changed through the implementation process?

*Complexity*

How does ROWTATE compare to usual care with regard to ease of use/delivery?

*Patient Needs and Resources*

How well does ROWTATE meet patients’ needs?

What are the barriers and facilitators to improving this?

*Design Quality & Packaging*

What did you think about the ROWTATE training and the manual?

*Cosmopolitanism*

How well does your organisation work with other organisations in the rehabilitation network?

*Networks and communication*

How does communication work in your organisation?

*Culture*

How would you describe the culture of your organisation with reference to ROWTATE? Do you think your organisation is good at learning how to do new things?

*Implementation Climate*

Do you think your organisation was in a good place to implement ROWTATE? Why do you think that?

Do you think ROWTATE was compatible with your organisation?

Was ROWTATE seen as a priority by your organisation?

Did you feel that there was a need to change practice in this area?

*Readiness for Implementation*

Do you think your organisation was fully committed to ROWTATE?

*Knowledge and Beliefs About the Intervention / Self-Efficacy / Individual Stage of Change? Other Personal Attributes*

How confident do you feel about delivering the ROWTATE intervention? How well does it work, in your experience?

*Other*

Is there anything else you would like to say about ROWTATE?

## Carer

Tell me about your relative, their accident and your involvement in their recovery.

How did ROWTATE work for them?

*Relative Advantage*

How do you think ROWTATE compares to the care usually provided in the NHS? Why?

*Complexity*

How does ROWTATE compare to usual care with regard to ease of use/delivery?

*Patient Needs and Resources*

How well were the needs of the person you care for met? What do you think are the barriers and facilitators to improving this?

*Culture*

How would you describe the employer/employing organisation of the person you care for?

*Implementation Climate*

Do you think the employer/employing organisation of the person you care for was in a good place to implement ROWTATE? Why do you think that?

*Knowledge and Beliefs About the Intervention / Self-Efficacy / Individual Stage of Change/Other Personal Attributes*

What do you think about ROWTATE?

How well does it work, in your experience?

How confident are you that the person you care for can carry out the actions that were recommended by the Occupational Therapist/Clinical Psychologist?

Is there anything else you would like to say about ROWTATE?

## GPs and AHPs

**Your role**

- What is your role and how do you usually support people with serious injury?
- What is your experience support people with serious injury return to work?
- How well do you think your support works helping people with serious injury return to work?

**Reactions to ROWTATE**

As part of this large programme of research we’ve developed a vocational rehabilitation intervention to help people return to and remain in work after discharge from a major trauma centre with serious injury. It involves occupational therapists and psychologists working together to identify and support people’s needs. The occupational therapists also work with and support the injured person’s employer (with their permission) to develop a return to work plan, advise on a phased return to work, and reasonable adjustments/ adaptations and modifications as necessary. It is delivered remotely in addition to people’s usual NHS rehabilitation.

- Have you heard of the ROWTATE study or intervention before this interview and if so what do you know about it?

If nothing, I will read you some information about ROWTATE for 1-2 minutes:

*The ROWTATE intervention commences within the first two weeks of injury. It is individually tailored to patient need and employment context in terms of content, dose, intensity and duration (up to 12 months). The intervention includes:*

*Assessing the impact of the injury on the participant, family and the participant’s role as a worker*

*Setting and reviewing vocational goals*

*Educating participants, employers and families about the effects of the injury and its impact on work and find acceptable strategies to lessen that impact*

*Monitoring and adjusting the participant’s post-injury life and work goals*

*Preparing participants for work by establishing structured routines with gradually increased activity levels and opportunity to practice work skills*

*Liaising with relevant stakeholders such as, employers, employment advisors (e.g. occupational health), solicitors and the healthcare team to advise about the effects of the injury and to plan and monitor a phased return to work*

*Routine monitoring of mood and emotional issues, via routine use of questionnaires, observation and responses during clinical sessions, by the OT.*

*Discussion with or referral to a clinical psychologist where needed. The clinical psychologist will deliver individualised psychological assessment and/or treatment and work with the OT the facilitate the patients return to work.*

- Given the information we have shared, what do you think about ROWTATE?
- How does ROWTATE compare to what you currently do:
- How easy do you think ROWTATE would be to implement alongside what you already do?

*Relative Advantage*

- How do you think ROWTATE compares to usual care? Why?

*Complexity*

- How does ROWTATE compare to usual care with regard to ease of use/delivery?

*Patient Needs and Resources*

- How well do you think your practice, and primary care more generally meets the needs of people returning to work after serious injury?
- What are the barriers and facilitators to improving this?

*Culture*

- How would you describe the culture of your practice? and the learning climate ?

*Implementation Climate*

- How would you find working with other professionals/organisations as part of an intervention such as ROWTATE? Why do you think that?
- Do you think ROWTATE would be compatible with your practice and with primary care more generally?
- Did you feel that there was a need to change practice in this area/more support for people with serious injuries returning to work?

*Knowledge and Beliefs About the Intervention / Self-Efficacy / Individual Stage of Change? Other Personal Attributes*

- What do you think about ROWTATE?
- How well do you think it would work in your practice?
- Are you confident that you/your organisation could help patients carry out the action recommended by the ROWTATE occupational therapist & clinical psychologist?

**Implementing ROWTATE**

- We are developing a toolkit to help implement ROWTATE in the NHS, so it would be helpful to know what information or resources would be useful for helping your organisation decide whether to commission ROWTATE?
- What are the major challenges you (as a GP, AHP, Practice nurse) see in implementing a programme like ROWTATE?
- What would convince you of its value?
- What would affect ROWTATE being implemented in your area?
- Who else would need to be convinced of its value?
- What is the best way of doing this? - What would convince them of its value?
  - Evidence of trial effectiveness – what types of evidence?
  - Information about trial efficacy – what types of information?
- What format for the above information? (Written/Event/Other)
  - What resources would you/your organisation need to influence uptake and implementation?

Is there anything else you would like to say about ROWTATE?

## Commissioners

**Your role and Commissioning**

- Who do you work for and what is your role in relation to commissioning?
- How does your organisation work in relation to others locally?
- What services do you commission to meet the needs of people returning to work after serious injury?
- How well do you think your organisation commissions services that meet the needs of people returning to work after serious injury?
  1. What are the barriers and facilitators to improving this?

**Reactions to ROWTATE**

As part of this large programme of research we've developed a vocational rehabilitation intervention to help people return to and remain in work after discharge from a major trauma centre with serious injury. It involves occupational therapists and psychologists working together to identify and support people's needs. It is delivered remotely in addition to people's usual NHS rehabilitation.

- Have you heard of the ROWTATE study or intervention before this interview and if so what do you know about it?

If nothing, I will read you some information about ROWTATE for 1-2 minutes:

*The ROWTATE intervention commences within the first two weeks of injury. It is individually tailored to patient need and employment context in terms of content, dose, intensity and duration (up to 12 months). The intervention includes:*

*Assessing the impact of the injury on the participant, family and the participant’s role as a worker*

*Setting and reviewing vocational goals*

*Educating participants, employers and families about the effects of the injury and its impact on work and find acceptable strategies to lessen that impact*

*Monitoring and adjusting the participant’s post-injury life and work goals*

*Preparing participants for work by establishing structured routines with gradually increased activity levels and opportunity to practice work skills*

*Liaising with relevant stakeholders such as, employers, employment advisors (e.g. occupational health), solicitors and the healthcare team to advise about the effects of the injury and to plan and monitor a phased return to work*

*Routine monitoring of mood and emotional issues, via routine use of questionnaires, observation and responses during clinical sessions, by the OT.*

*Discussion with or referral to a clinical psychologist where needed. The clinical psychologist will deliver individualised psychological assessment and/or treatment and work with the OT the facilitate the patients return to work.*

- Given the information we have shared, what do you think about ROWTATE?
- What do you think about ROWTATE?
- How well does it work, in your experience? / How well could it work?
- How does ROWTATE compare to usual care?
  1. Ease of use/implementation
  2. Delivery mechanisms
  3. Outcomes
- Do you feel that there is a need to change or improve the way we support people back to work after long-term injury?
- Does / could ROWTATE meet that need?

**Future Commissioning Challenges**

- What are the pressing local health priorities commissioning in your area is seeking to address?
- Would your organisation consider ROWTATE a suitable model for commissioning?
  - If yes, why
  - If no, why not and what could we do to change this?
- Is there anything about ROWTATE that is not a good fit with the way health services in your area are commissioned and delivered?
- What are the major challenges you see in commissioning a programme like ROWTATE?
- What would affect ROWTATE being commissioned in your area and convince commissioners of its value?
  - 1. Evidence of trial effectiveness – what types of evidence?
    2. Information about trial efficacy – what types of information?
    3. What format for the above information? (Written/Event/Other)
- What resources would your organisation need to influence uptake and implementation?
- We are developing a toolkit to help implementation of ROWTATE in the NHS, so it would be helpful to know what information or resources would be useful for helping your organisation decide whether to commission ROWTATE?
  1. Evidence summary
  2. Return on Investment Tool
  3. Business case
  4. Real-life case studies from ROWTATE participants
  5. Video for commissioners describing ROWTATE, its effectiveness, cost effectiveness and acceptability to patients and therapists
  6. Implementation Gantt chart
  7. Service specification
  8. Example delivery models
  9. Logic model (diagram detailing inputs, activities, outputs, mechanisms, outcomes)
  10. Suggested monitoring process and monitoring tools
  11. Suggested evaluation methods and evaluation tools

Closing: Any other thoughts about ROWTATE?
