# Supplementary material for: Implementation barriers and facilitators to a vocational rehabilitation intervention after traumatic injury (ROWTATE) in the UK: qualitative interviews with key stakeholders
Source: BMJ Open. 2026 Jul 30;16(7):e118198. doi: 10.1136/bmjopen-2026-118198 (PMC13422936; doi:10.1136/bmjopen-2026-118198)
Supplement: online supplemental file 2 [file bmjopen-16-7-s002.docx]

| **Domain 1: research team and reflexivity** | | |
| --- | --- | --- |
| **Personal characteristics** | | |
| 1. Interviewer/facilitator | Which author(s) conducted the interview or focus group? | Multiple researchers carried out the majority of interviews including experienced researchers (RL, CM, BK) and one PPI group member (SF) |
| 2. Credentials | What were the researcher’s credentials? (e.g. PhD, MD) | CM and BK each have a PhD. RL and SF are lay researchers. |
| 3. Occupation | What was their occupation at the time of the study? | CM was a senior research fellow, BK was an Associate Professor, RL was a research assistant and SF was PPI lead. |
| 4. Gender | Was the researcher male or female? | CM, BK and RL – female,  SF - male |
| 5. Experience and training | What experience or training did the researcher have? | CM, BK and RL were experienced qualitative researchers with health services and implementation science backgrounds.  SF was a trained PPI contributor who co‑interviewed some participants. |
| **Relationship with participants** | | |
| 6. Relationship established | Was a relationship established prior to study commencement? | No prior relationship was established between the researchers and participants |
| 7. Participant knowledge of the interviewer | What did the participants know about the researcher? (e.g. personal goals, reasons for doing the research) | Participants knew the research purpose, organisation, roles, and had contact with a researcher to book interviews. Participants received an Information sheet, consent form and details on study purpose (before interview). |
| 8. Interviewer characteristics | What characteristics were reported about the interviewer/facilitator? (e.g. bias, assumptions, reasons and interests in the research topic) | Researchers had experience in trauma, VR, and implementation science. BK, RL and SF had previously worked in rehabilitation research. A PPI co‑interviewer contributed to inclusivity. |
| **Domain 2: study design (Methods)** | | |
| **Theoretical framework (Methods)** | | |
| 9. Methodological orientation and theory | What methodological orientation was stated to underpin the study? (e.g. grounded theory, discourse analysis, ethnography, phenomenology, content analysis) | Two‑stage analysis: framework coding (CFIR, TFA), followed by reflexive thematic analysis.  (Page 6) |
| **Participant selection (Methods / Results)** | | |
| 10. Sampling | How were participants selected? (e.g. purposive, convenience, consecutive, snowball) | A purposive sample representing the trial stakeholders. Recruitment pathways differed by stakeholder group. Patients and therapists were invited via the RCT. Employers, GPs and commissioners were recruited via patient contact or professional networks due to low direct response.  (Page 6/7) |
| 11. Method of approach | How were participants approached? (e.g. face to face, telephone, mail, e-mail) | Participants were emailed letters of invitation, an information sheet and a consent form. (Page 6) |
| 12. Sample size | How many participants were in the study? | We interviewed 80 participants across stakeholder groups (Page 7) |
| 13. Non-participation | How many people refused to participate or dropped out? Reasons? | Refusals not formally recorded; employer and GP participation was low due to limited patient consent for employer involvement and low response rates from GPs, |
| **Setting (Methods/Results)** | | |
| 14. Setting of data collection | Where was the data collected? (e.g. home, clinic, workplace) | Interviews took place online using MS Teams or by telephone. (Page 6) |
| 15. Presence of non-participants | Was anyone else present besides the participants and researchers? | Only the researchers and participants were present. |
| 16. Description of sample | What are the important characteristics of the sample? (e.g. demographic data, date) | See Table 1 for interview groups  (Page 7) |
| **Data collection (Methods)** | | |
| 17. Interview guide | Were questions, prompts, guides provided by the authors? Was it pilot tested? | Topic guides with prompts were co‑produced with PPI and are included in supplementary material. These were piloted in our feasibility study. |
| 18. Repeat interviews | Were repeat interviews carried out? If yes, how many? | Some patients and therapists (n=13) were interviewed twice (acceptability and implementation); others only once depending on availability. |
| 19. Audio/visual recording | Did the research use audio or visual recording to collect the data? | Interviews were audio-recorded and transcribed verbatim (page 6) |
| 20. Field notes | Were field notes made during and/or after the interview or focus group? | No |
| 21. Duration | What was the duration of the interviews or focus group? | Interviews ranged in duration from 10 to 55 minutes |
| 22. Data saturation | Was data saturation discussed? | Saturation not used; sampling guided by information power |
| 23. Transcripts returned | Were transcripts returned to participants for comment and/or correction? | Transcripts were not returned to participants |
| **Domain 3: analysis and findings (Methods)** | | |
| **Data analysis** | | |
| 24. Number of data coders | How many data coders coded the data? | Three researchers (CM, RL and BK) independently coded the data (Page 6) |
| 25. Description of the coding tree | Did authors provide a description of the coding tree? | Coding organised within TFA and CFIR frameworks; no hierarchical coding tree produced (consistent with reflexive thematic analysis) (page 6) |
| 26. Derivation of themes | Were themes identified in advance or derived from the data? | Themes inductively generated from data following framework coding; not predetermined. (page 6) |
| 27. Software | What software, if applicable, was used to manage the data? | NVivo 15 (page 6) |
| 28. Participant checking | Did participants provide feedback on the findings? | No |
| **Reporting (Results – Acceptability Data)** | | |
| 29. Quotations presented | Were participant quotations presented to illustrate the themes/findings? Was each quotation identified? (e.g. participant number) | Quotations have been presented throughout with participant codes assigned and used against quotations (Pages 8-13) |
| 30. Data and findings consistent | Was there consistency between the data presented and the findings? | We endeavoured to report the study findings in a clear, consistent manner in order to accurately reflect the data that have been collected.  Yes, themes are clearly presented.  (Pages 8-13) |
| 31. Clarity of major themes | Were major themes clearly presented in the findings? |  |
| 32. Clarity of minor themes | Is there a description of diverse cases or discussion of minor themes? |  |
