# Supplementary material for: Implementation barriers and facilitators to a vocational rehabilitation intervention after traumatic injury (ROWTATE) in the UK: qualitative interviews with key stakeholders
Source: BMJ Open. 2026 Jul 30;16(7):e118198. doi: 10.1136/bmjopen-2026-118198 (PMC13422936; doi:10.1136/bmjopen-2026-118198)
Supplement: online supplemental file 3 [file bmjopen-16-7-s003.docx]

| **CFIR constructs** | **Themes and Sub-themes** | **Facilitators to the ROWTATE intervention delivery, coded by CFIR components (source of data)** | **Barriers to the ROWTATE intervention delivery, coded by CFIR components (source of data)** |
| --- | --- | --- | --- |
| 1. **Innovation** | ***The “thing” being implemented*(Curran, 2020)*, e.g., a new clinical treatment, educational program, or city service.*** | | |
| **The innovation is often complex and multi-faceted, with many interacting components (Butler et al., 2017). Innovations can be conceptualized as having ‘core components’ (the essential and indispensable elements of the innovation) and an ‘adaptable periphery’ (adaptable elements, structures, and systems related to the innovation and setting into which it is being implemented) (Fixsen, 2007; Greenhalgh, Robert, et al., 2004).** | | | |
| ***1a) Source*** | **Patient Needs & Outcomes** | *We [the UK] seem to be actually world leaders in rehabilitation (EMP02)* | *We’re years behind in terms of the policies being brought in. (EMP02)* |
| **1b) Evidence base**  The innovation has robust evidence supporting its effectiveness. | **Patient Needs & Outcomes** | *It was nice, the reassurance and the fact that people care about me and asking me if I’m okay (PA01)* | *“ think it’s the wrong outcome…* *the trouble with return to work as an outcome… is we are not in control of that. So if you had a traumatic accident now, or I do, and develop severe epilepsy and I’m a taxi driver, no amount of exceptionally good vocational rehab is going to get me back to work.* *(ME01)* |
|  | **Buy-In** | *So any quality research I believe would need to actually not just headline the research, but actually convert it into an HR policy, which is in effect – almost like the Ikea thing, it’s on the shelf, ready to go. (EMP02)* | *I think there's something about what, what, what our end goals are, what success really means. Understanding that obviously there's a cost, there's a wider cost to this on understanding the impact and that's where I think kind of working with the kind of wider teams, local authority really understanding those wider, why there are aspects that economical growth for the for the system, which is part of our mission as a, as an ICB, is to kind of improve that economic growth.* (COM02) |
| ***1c) Relative Advantage***  The innovation is better than other available innovations or current practice. | **Employer Engagement** | *So, they would never have gone to the level that [the occupational therapist] did in terms of the one to one care, advocating for [the employer]. So, either we’ve got a very bad occupational health provider, or my experience of them have been, but I think we definitely got more richness from that interaction with the [ROWTATE] service.* *(EMP01)* | *It can be really hard for people to have those conversations themselves with the employers, and for the employers sometimes to have those conversations, for all kinds of reasons. People can kind of over report or minimise some of the difficulties that they have as an employee, and there’s some concern about how ultimately that they don’t want to be letting down the people that they work with, their employers. There’s often some masking that can go on there around difficulties that they’re experiencing. (TA11)* |
|  | **Mentoring** | *It’s nice to have the mentoring sessions. I think they’re really helpful.*  *I think just helpful to have someone to talk to if you’re not sure about something. And, you know, especially people with experience.. We’ve got enough support because you’ve got a good structure as well that you can follow*. (TA02) | *If you’re mentoring people come up with problems and you think you know the answer but you as a mentor want to check it out to make sure you’re telling them right and everybody’s doing the right thing, so I think having that support as a mentor and knowing who to go with queries [is important]* *(ME01)* |
|  | **Patient Needs & Outcomes** | *It could help everybody concerned much quicker than the conventional route* (EMP04)  *It’s almost as if when you walk out of the hospital the medical staff wash their hands of you and that’s it.. The only person I had contact with was [my occupational therapist], she was phoning up. And for me, that was a comfort blanket, that was fantastic. (PA05)* | *Sometimes there seems to be a quite a smooth, clear path and people have had physio input or whatever else they might need and that's gone according to plan and there's a process in place. I think other times it's a lot more messy and complicated, and I guess those are probably situations where clearly it then becomes a lot more challenging and probably where the support for getting people to return to work is more lacking essentially. (GP01)* |
| ***1d) Adaptability***  *The innovation can be modified, tailored, or refined to fit local context or needs.* | **Employer Engagement** | *I can see the benefits for both organisations, for small and large. Because the small ones, if they’ve got a smaller team, then somebody being out of that team through injury or trauma is a bigger impact. But then the larger organisations are more likely to have wellbeing initiatives and engage in employee assistance programmes, and so it could just be part of their armoury of support in certain situations. So I think that there’s an appetite in all sizes or organisations, and all sectors as well. Yeah, I think it’s invaluable for all employers really, and employees.* (EMP04) | *I think essentially, it sounds dangerously compatible only with very big organisations (EMP02)* |
|  | **Buy-In** | *Depending on the culture of the NHS Trusts and the local – you know, what the local context is like, it might sit nicely in acute, and in other places it might sit better in community.* *I think it’s about having that flexibility. As long as there is an understanding of, this is how the intervention works, where it’s sited, or where the OT comes from and psychologist comes from probably doesn’t matter so much. (ME02)* | *So I think you really need to sort of say this, this is the structure, but you can't be rigid on that structure because the NHS you put in steak and out comes a sausage. You know it's, you know, it's always going to be a compromise. And I I was involved in, they tried to replicate Maud Graffs work around supporting people with dementia in the early stages in Holland, and they tried to. Wenborne was replicating it in the UK and it didn't work, and it's because the NHS can't take a pure model and replicate it. So they didn't see the impact that they saw in the Netherlands. So I think you just have to say that you need a combination of this experience, but allow them to decide how they're going to do that. (COM07)* |
|  | **Patient Needs and Outcomes** | *She said if there was any problems that, you know, feel free to call her at any time.* (PA01)  *I also like that it is tailored to each person’s needs* (TA01) | *I think I’d have done more physical stuff had I been face-to-face with the person. But, in general, I don’t think it’s been a problem. I think everyone has managed to rehab, despite that, and have managed to get back to work so far. Then also, with that kind of physical side of things, that’s why we had the community OTs and physios anyway. (TA09)* |
|  | **Remote working** | *I like giving patients the choice because factors like if they are working or if they’ve got a long travel, we see people out of area as well, so the benefit of remote working and the convenience for patients can be really high. (TA04)* | *I wouldn’t ever want to only offer remote without there being an option for face-to-face, and I guess with ROWTATE the default is more remote rather than giving the patient the choice. (TA04)* |
| ***1e) Trialability***  The innovation can be tested or piloted on a small scale and undone. | **MDT** | *It probably ended up that I was spending much more time on ROWTATE at the feasibility stage than I planned to, and that probably led to some changes in some of the ways we assess, kind of looking at over a longer period of time we might keep people in an active monitoring, watchful waiting phase, to sort of see, actually, you know, is clinical psychologist input warranted (TA11)* | *I’ve never done research before, so this process is all completely new. So yeah, definitely, if it was just focusing on the intervention stuff, yeah, I think it would be a little easier. (TI03)*  . |
| ***1f) Complexity***  *The innovation is complicated, which may be reflected by its scope and/or the nature and number of connections and steps.* | **Employer Engagement** | *I would say that this service provides an invaluable support for employers and employees navigating very complex cases of getting an employee back into the the workplace. And without it, some of it is a bit, ‘gut feel’ for employers, and I come back to one of my earlier comments about I am not a medical professional and I will never pretend to be and so having medical professionals that can give honest and open opinions that an employer can make conscious choices based on all information rather than from the employee or their own perspective, I think is incredibly important because if it does come from the employee, it is always going to be slanted towards where they are psychologically, what they want to influence and what they want it to look like. But when you have that third party that's working with you so closely, it can give a really informed lay of the land for decisions to be made, both for the benefit of the employer and the employee. (EMP06)* | *I think one of the biggest barriers are what I would call a frictional barrier or a transactional barrier. Because most employers, and most patients in effect, do not know how long their rehabilitation will take, and so as a consequence of that, employers don’t know how long they’ll take to learn what they don’t know (EMP02)* |
|  | **Buy-In** | *I think there's always probably more to do in terms of this, but it's recognising that it's, there's the complexity both across our places to deliver that, but also the complexity of the kind of needs that will be required for patients with the following such injuries. (COM02)* | *It's health and care and it's working with other local authorities and getting people back to work is very much about working with our local authorities there. So sometimes they're barriers there as a result of that, and that's part of the reason for ICB's is to try and break down some of those barriers and work closely with our local authorities and partners (COM02)* |
|  | **Patient Needs & Outcomes** | *I’ve had quite a few patients who’ve had questions about their immediate care following discharge from hospital, so I would contact our local major trauma team, who are meant to be following up, and say, “Oh, Participant X, Participant Y, have these questions, queries and concerns. When you’ve got time, can you give them a ring to contact them?” and they’re like, “Why are you asking us to contact them? They can ring themselves.” And I’ll say, “Well, they’ve rung themselves and not had any luck, and they’ve asked me to explain in more detail exactly what it is “ (TA09)* | *And they were like, “Yeah, but we can’t see your electronic records, we don’t know anything.” And I’m like, “But that’s not my fault, I still need your help. (PA08)* |
|  | **Patient Needs & Outcomes** | *Often they’re of working age and I know through experience that even though it might seem – even if people might think it’s straightforward, it never is, you know what I mean? And people seem to benefit from the support of having somebody to help just steer them through, and the employer seem to value that as well because they’re scared. (ME01)* | *I think the patient needs to speak to someone and talk about what they’re going through. It’s really good for the recovery. I think that did help the recovery. I think she got a bit too dependent on them. (CAR01)* |
| ***1g) Design***  The innovation is well designed and packaged, including how it is assembled, bundled, and presented. | **Patient Needs & Outcomes** | *Well, it was good because it started so quickly. I don’t know if it was a couple of days after I’d come home or a week after, it wasn’t very long anyway, so from that point it’s like getting into that mindset of how I’m going to get back to work because in the early days I didn’t know if it’d happen.(PI03)* | *I don't know, in some instances, I wonder if it’s too much for the person. I think in the situations where someone is in hospital.., I think that’s stressful for them. And then someone phoning and trying to find out about what their job is, they’re just thinking, I don’t even know if I’m going to walk again (TA01)* |
|  |  | *I’ve never been in this situation before, it’s hard to come up with an idea of how you go about it (PA06)* | *I’ve had a couple where I think they probably haven’t initially needed it, in the sense that they’ve got really good, supportive employers and the employers are sort of putting in everything I’m recommending anyway, and it’s probably been a duplicate service that they haven’t really needed. I’d say I’ve got a handful of those. (TA02)* |
|  | **MDT** | *I thought that was something that ROWTATE offers really well, because half of my participants needed psychological input. Even from me, coming from the acute setting, that’s something that we never even really consider, yeah, we know that they’ve had a traumatic accident, and you tell them that, “Oh, you will be feeling a bit emotional for a while, but it will get easier,” but then we’re six months down the line with these ROWTATE patients, and they’re still needing so much psychological input, and they’re really benefiting from it. (TI13)* | *I don’t know if I really picked up at the training that you wanted us to work together with psychologists. I felt it was more, well, we’ll be ticking along, we’ll do our wellbeing questionnaires, we’d see if there was caseness, then we would refer on. I suppose I didn’t pick up that desire for us to have joint sessions. So that’s quite interesting, reflecting on that. (TI08)* |
| ***1h) Cost***  The innovation purchase and operating costs are affordable. | **Employer Engagement** | *From an employer’s perspective, let’s face facts, people are running a business to sell their products and services and to make money and to crack on with that, and to have someone who’s key to their business out of the loop for whatever period of time, indefinitely, is a challenge to business owners, no matter how much they love the employee, is it still a challenge, they’ve got a hole there that they need to fill (EMP04)* | *I think, like there are standard software tools like Sage, which is a standard software tool for running a business, knowing what your business costs are, what your outgoings are, what your income is from sales, there are standard tools for doing it. I think there should be some additional web pages with what the evidence base from ROWTATE would be. (EMP02)* |
|  | **Patients Needs & Outcomes** | *I wouldn’t call it a cost. Yes, it does take a bit of time, but it’s not like you're not receiving something from it. (PA04)* | *I honestly think like it would be so good if you could provide this to everybody, but my concern is that you couldn’t provide it to everybody because it’s very cost-heavy, time-heavy, all of that sort of thing. (PI12)* |
|  |  | *Otherwise I would have been contacting my GP, because I wasn’t going as fast as I thought I was going, or contacting the hospital, because I had the number for the hospital, but actually if that was the right level I was at then – but if I hadn’t spoken to [TO24], I wouldn’t have had that knowledge, and therefore I’d be using more of the NHS time, which was probably unnecessary. (PA14)* | *I just can't see at the moment, the way the money's funded, how. I think you could get it commissioned for a period of time, but it's rolling it out and getting it adopted and more widespread (COM07)* |
| 1. **Outer setting** | ***The setting in which the Inner Setting exists, e.g., hospital system, school district, state.*** | | |
| ***Project Outer Setting(s): UK NHS Health System, UK Community health setting, UK social care system, UK welfare system, UK employment system*** | | | |
| **2a) Critical incidents**  Large-scale and/or unanticipated events disrupt implementation and/or delivery of the innovation. | **Remote** | *I think what made the difference.. was COVID. So from previous ways of working that were fairly kind of myopic in their vision as to what that should look like, and you bring people in to a clinical space and you book your room and you have your therapy sessions for 50 minutes etcetera. …. I think what has happened is that you know through lockdown and what that's meant is that most organisations have had to find ways of working through that. And so, you're probably better positioned to roll something like this out now than. It certainly would have been 5-5 years ago. (ME03)* | *COVID was going through [NHS Hospital Name D] like a dose of salts, so they were like, “If you're fit and can literally limp and you get out of the building, you will go.” So I ended up out probably quicker than I would have been (PA08)*  *I think in the beginning it was quite difficult to get started, and I think COVID did impact on how quickly we could kind of get up and running. (TA04)* |
| **2b) Local attitudes**  Sociocultural values (e.g., shared responsibility in helping recipients) and beliefs (e.g., convictions about the worthiness of recipients) encourage the Outer Setting to support implementation and/or delivery of the innovation. | **Employer Engagement** | *We want to have that feeling of connection, caring for our employees, and we’re putting a lot of resource behind doing that. So, that’s what we stand for. (EMP01)* | *For the majority of employers, they’ve got a business to run, they’ve got work to crack on with, and so unfortunately my experience is that quite a few employers are light touch, they pay lip service to wanting to be supportive and ticking the box to make sure that they’re not going to get sued for disability discrimination (EMP04)* |
|  | **Mentoring** | *I think my role as a mentor is to give the OTs confidence that they can do it, and … it’s about giving them confidence, knowledge and the space to explore how they do it and ask questions. I think it’s about giving them space to ask questions and then helping others (ME01)* | *So there’s lots of stuff that we talk about in this mentoring that would never get talked about if it was rolled out in the NHS. So it would make it – what I’m trying to say is, it wouldn’t necessarily need to take up quite as much time maybe, and it could perhaps run in the background, a little bit more, with the use of technology (ME02)* |
|  | **MDT** | *Where it’s worked really well is then the complete opposite to that, so where the OT and the clinical psychologist are both initiating these communications, it’s not coming more from one than the other, and they’ve having regular times where it’s just them, like little mini MDT meetings. And they’re getting to, you know, “She did this with me,” “Oh yeah, she did that with me as well, and I did this, and that worked.” So solving problems for the participant, and doing that very successfully, and managing some very, very difficult situations that participants bring into this rehabilitation pathway. So it’s open communication, it’s being open to what the other person’s role is and trying to really understand that. And seeing how you can mesh together as a team of two, to deliver the intervention, for the benefit of the patient, participant. So that’s where it’s worked really well, and I’ve had some stories both from clinical psychologists and from OTs demonstrating great joint working,*  *(ME02)* | *In the early days, I had no idea why some of the clinical psychologists were just not answering the OTs, when they were making referrals, or trying to communicate with them. I didn’t really understand how they might want to build up their caseloads, did they only want one or two participants? Did they want ten or eleven participants? You know, what would be seen as normal? Whereas I had that sense with the OTs. (ME02)* |
| **2c) Local conditions**  Economic, environmental, political, and/or technological conditions enable the Outer Setting to support implementation and/or delivery of the innovation. | **Patient Needs and outcomes** | *I feel that the patients who have engaged have appreciated that somebody was in touch with them, because they’ve had very limited access with other NHS professionals. So I think I provided a lot of reassurance. (TA01)* | *Some of them that have got really good support at work already aren’t really needing the intervention and there are – but there are also many, especially if they haven’t got good support at work, that really do. (TA02)* |
|  | **Employer engagement** | *I’ve got the knowledge of doing this certain job… and now I am the only one (PA01)* | *The sad fact is it depends how valuable their role is to the company. (EMP04)* |
|  | **Remote** | *I thought it worked quite well really. I mean considering, like we say, considering that Covid was around, I think it worked quite well. (PA01)* | *So there’s an understanding of the local context that’s more comprehensive and therefore useful to help the person through this complex pathway, than having two people that are completely remote, and do their very best, but have no idea what happens in [other areas] when they’re based up in somewhere else in the country. (ME02)* |
| **2d) Partnerships**  The Inner Setting is networked with external entities, including referral networks, academic affiliations, and professional organization networks. | **Buy-In** | *This should be linked to the access to work programme (EMP02)* | *It can be very interesting, but yes, you've got different ICBs that have different priorities, different agendas, different processes. Different ways of working, some of which you know, work very, very much in tandem. Sometimes it can feel a little bit not quite that. (COM04)* |
|  | **Employer Engagement** | *Occupational health comes along and they won’t be aware of that history in the same way and probably from an employee perspective are seen very much as something the employer is doing there, rather than it being the support for them. (EMP05)* | *There’s a lot of interdependencies with the relationship between the colleague and their medical practitioners, so whoever that is. So, we are reliant on them to link back to us and we’re very willing to talk to people. That’s where I think you are needing the support much more. (EMP05)* |
|  | **Patient Needs and outcomes** | *Some of [my problems] could be down to my GP and maybe the complexity of all the stuff that was happening, with so many different people involved in my recovery. My [Occupational Therapist] and [Clinical Psychologist], they were the lion’s share of that stakeholder support of mine… because the others didn’t pick it up I suppose in a way. (PA08)* | *If somebody's got a really serious injury that's obviously managed in secondary care and then the patients are discharged, and we'd expect them to have follow up in place for what they needed. You know, and that OT needs and physio needs and psychology needs would come under that. So obviously we can look after the general practice related needs of somebody who's had a serious injury. (GP04)* |
| **2e) Policies and Laws**  Legislation, regulations, professional group guidelines and recommendations, or accreditation standards support implementation and/or delivery of the innovation. | **Buy-In** | *So we know that you know, global population health people are living for longer with health conditions, multiple health conditions and in the UK, we're seeing a real rise in that...But what we're finding is healthy working life is not matching length of life, so it is an area of concern. And we focused on the fact that with the last government there was consensus agreement across all political parties that this had impact and you started to hear about economically inactive concerns around productivity and a particular focus on people with ongoing health conditions. (COM07)* | *There was a return to work programme in about 2008, run between the Department for Health and the Department for Work and Pensions (EMP02)* |
|  | **Employer engagement** | *What was quoted to me from HR is they’re expecting things like either critical illness cover or whatever else. So, the company does provide that the insurance, in inverted commas, to cover that, but it doesn’t. (EMP03)* | *So what happens is when something like this happens, they reach for the only policy document they’ve got, which is the standard redundancy manual, and then they negotiate them out of the workplace. (EMP02)* |
| **2f) Financing**  Funding from external entities (e.g., grants, reimbursement) is available to implement and/or deliver the innovation. | **Employer Engagement** | *Some of the other employers do have the luxury of being able to support their employees, and have medical insurance in place for example (EMP04)* | *The small employers perhaps are wanting to get somebody off their books, because they can’t keep the job open indefinitely* *It costs money to be a good employer* *(EMP04)* |
|  | **Buy In** | *I don't know whether it’s something that you could speak to insurance brokers about, like Vitality, to tag it on, that this is – you know, like, for example when I get the policy schedule and it says all about, you know, if I’ve got cancer then as soon as I’ve got this diagnosis I’ve got all this support. (EMP04)* | *So the usual – funding, money, access, they're all huge barriers to things .. (GP02)* |
| **2g) External pressure**  External pressures drive implementation and/or delivery of the innovation. | **Employer Engagement** | *I want to know is he safe, can he do his job? And if he can’t, that’s fine. What do we do? What reasonable adjustments do I need to put in place? (EMP03)* | *And they have a business to run, you know what I mean? It isn’t return to work at all costs. You have to return a patient that is able to do the job.(ME01)* |
|  | **Buy In** | *Well, the natural thing would be through the access to work programme, the extension of access to work. (EMP02)* | *They are, as every Council is going through, really big financial pressures at the moment. They're kind of, they're kind of in a bristle right on the edge of kind of bankruptcy position as a result, that's really straining relationships. They're looking to push back on everything to save money and that's making it really difficult. (COM03)* |
|  | **Mentoring** | *I don't know how much supervision and support people get in their day jobs anymore, and I think particularly in a new role or something that’s a little bit different, I think it’s a very worthwhile thing. (ME04)* | *If you're factoring in an hour appointment, maybe a bit longer if it’s a couple of appointments split, plus the time to document all that appropriately, and then the mentoring, receiving mentoring on a monthly basis, that’s kind of where I was estimating it, that I’d have a half day-ish per week to dedicate to ROWTATE. So that definitely got stretched around that time where I had the caseload of ten. (TA11)* |
| 1. **Inner setting** | | **Inner Setting: The setting in which the innovation is implemented, e.g. hospital, school, city.** | |
| **Project Inner Setting(s): ICB localities, NHS Trusts, Hospitals, Primary Care, Community providers, homes, employment sites** | | | |
| **3a) Structural characteristics**  Infrastructure components support functional performance of the Inner Setting.  *(Physical, IT, Work organisation)* | **Employment Engagement** | *Understanding the time frame….when you’re in the thick of it, it was a real gap to us. (EMP01)* | *It’s a year, and how do you then hand it over and who do you hand it over too? What would I want to see, to expect? I would expect ROWTATE to last as long as the physical side of it is because he’s still going for scans and things. That bit hasn’t ended yet. Of course, in the background, there’s solicitors around the claim that’s going in with this as well, BUPA, the company obviously health provider, that’s racked up money. They’re very good at that (EMP03)* |
|  | **Employment Engagement** | *The larger organisations are more likely to have wellbeing initiatives and engage in employee assistance programmes, and so it could just be part of their armoury of support (EMP04)* | *Sometimes our HR team are just, they’re quite young and they’ve read the book…dummies guide to HR [but]..they’ve not dealt with and led people. (EMP03)* |
|  | **Employment Engagement** | *It was a joint discussion I think, between myself and the HR people. (PA09)* | *The procedure is very mechanical….It triggers all these things for you to do. (EMP03)* |
|  | **Remote** | *I did one from a caravan. And I didn’t want to not do it, and I was very pleased with my IT skills. (ME01)* | *I know if it was my mum she wouldn’t, she’d never do a video call (PA03)* |
|  | **Mentoring** | *I had quite a large case load… I've reduced that to make space for the mentoring, so it's kind of kept it bounded within time that I devote to ROWTATE, but just sort of balanced within that. (ME03)* | *Let’s say it was an intervention that got incorporated into existing teams or existing services, or things like that, I think there would need to be opportunity for therapists delivering that intervention to have dedicated time to talk about that intervention, and share with each other I think. (ME04)* |
| **3b) Relational connections**  *There are high quality formal and informal relationships, networks, and teams within and across Inner Setting boundaries (e.g., structural, professional).* | **Employer Engagement** | *[Supporting the employee] was easy…, I think because the relationship I had with [the employee], not.. how it could have if it was someone else in my organisation, put it that way. (EMP03)* | *I’m waiting for someone to do an appointment for X, Y, Z and then he’s waiting for that appointment to come along, while seeing someone else, so again, whether that’s clinical physician, or whether that’s occupational health, and if I’m brutally honest, none of them are aligned. Even our internal occupational health said, you need to have a capability assessment team. I’m like yeah, I get that. You need a physio, you need this, you need that, and I asked them, who is doing that then? Oh, that is your responsibility as a line manager. (EMP03)* |
|  | **Mentoring** | *I see us as being all one big department, they probably don’t see that. But because I get to know nearly all of them at one stage or another, and I’m not mentoring the CPs, but I hear about what they’re doing, via the OTs, then I get to hear what kind of a clinical psychologist they are as well. So I get to know all of them, and some of them don’t know that I know quite a lot about them, so it’s (laughs) – so it’s all nice really, it all feels like a big – I won't say happy family, but it feels like a nice big team, rehab team. (ME02)* | *I think maybe the joint sessions with the participant, it’s a good idea, but I think timing wise, it might be tricky to organise that. Because at the moment I’ve got a flexible role so I can do mornings, that tends to be better for participants from my experience. Whereas the CPs that I work with, often they’re doing it in the evenings, which I couldn’t do. So I think maybe for logistics, that might be tricky. (TI06)* |
|  | **Patient Needs and Outcomes** | *She was able to offer lots of good advice about how to influence my GP, who was being nonresponsive and not accepting I’d had an accident. So it was very good to be able to talk to somebody who understood the literal pain I was in, and the confusion and complications of not having a GP who would acknowledge I needed their support. So that was really good. (PA08)* | *There are so many variables because you're .. dealing with people involved in that person’s job, their employer, HR, oc-health, GPs, consultants, other employees (ME04)* |
|  | **Buy In** | *Now working as the ICS, it’s public health, but then pulling in NHS partners, and it is about what can we do as the NHS. And sits alongside the programmes that we call as an anchor institution, how can we support our local communities.(COM01)* | *We kind of talk about and we struggle with this concept of what that we call 1/3/6 and then more… So the 1 being us, the 3 being our local authority footprints and the six being our localities, and we don't always have a joined up approach, we don't. Well, I don't think we're all on the same page yet around what we do at each level. (COM03)* |
| **3c) Communications**  There are high quality formal and informal information sharing practices within and across Inner Setting boundaries (e.g., structural, professional). | **Employer engagement** | *I think just opening that channel of communication enables people rather than disables people. Quite often, they’ve either got their head in the sand and they don’t know where to start, or there’s vulnerabilities with the employers as well, “We don’t want to make somebody worse.” So, often the employers can delay things that don’t need to be delayed because they don’t have an up to date story about the person. (TA10)* | *[Patients] are liaising with their employers themselves and sort of translating some of the stuff that we’ve done through them. My lady who I did the ergonomic workstation assessment for, she just wanted the report written to her and then she was going to share that with her employer, she didn’t want me to send that to her employer directly. So I think it’s just they wanted to have ownership of that relationship between themselves and the employer when it came to returning to work. (TA03)* |
|  | **Patient Needs and Outcomes** | *Definitely an emphasis on very much communicating, not just to the participant, but communicating with other professionals and being proactive in that, rather than waiting for someone to ask for the information, to really take the lead I suppose is the difference. (ME04)* | *Every time I went to the GP, it was like, “This is my discharge paperwork, it says I’ve been hit by a car, it says I’ve left on Oramorph and everything, and it says handover to GP,” but yeah, they wouldn’t take it. They were like, “Oh, they’ll write to us.” And I was like, “Well, what’s the point of waiting for them to write? I’m sat here now,” (PI08)* |
|  | **MDT** | *Some of the best sessions that I’ve had that I think have been really useful as well for the participants have been those where it’s been a joint session between the OT and the clinical psychologist. And you really get that coordination in the room there, where we’ve both had our perspectives from individual sessions perhaps but we come together and we have this kind of shared discussion around, okay, so what are the goals now? What are we working towards? Who’s going to pick up what? With the participant there as well to be at the centre of that.(TA11)* | *Where it is difficult is – and then it’s not every psychologist, by any means – if you email somebody because a participant has asked you to and you don’t get a response until a week later, and you email back but then you don’t have any response. (TI08)* |
|  | **Remote** | *I think, on the whole, in terms of engagement and establishing relationships, in my opinion, I managed to do that virtually, in the same way I would face-to-face. I have had patients that have broken down crying and I don’t feel like the virtual side of things has negatively impacted on that. I feel like I was able to give them the same support I would as if I was face-to-face (TA06)* | *I can imagine that would be quite hard if you were a floating psychologist and not kind of embedded in the different teams. In a sense it could be harder, but then actually all of the interactions that I have with members of the team here in [AREA] are all online via MS Teams so I think there shouldn’t be too much of a barrier with that even if you are a floating OT or psychologist. (TA04)* |
| **3d) Culture**  There are shared values, beliefs, and norms across the Inner Setting. | **Employer Engagement** | *I think it wholly fits with the values of our organisation. It provides an extra level of care for employees and it helps the organisation think about that employee's journey back to work in the in the right way and the healthiest way for the employee.(EMP06)* | *The private sector will be more difficult than the public sector, and there always tends to be a natural focus on the public sector. But because the public sector has got the public sector equality duty, it’s got additional responsibilities, so you’d expect the public sector to be the best. But that means then in terms of what you prioritise or where the procedures are evidence based to be of most use, it’s got to be in the private sector I think. (EMP02)* |
|  | **Buy In** | *I mean, it definitely would demonstrate return on investment. I think it’s that – what we would end up having to demonstrate is how do we – what do we stop doing or what do we do differently as a result of ROWTATE? And that’s where there’s a big focus on how do we do things more efficiently, how do we do things differently, we’re not in a place where we can just continue to do the same thing. (COM01)* | *I don't know our statistics for our area, which is interesting in itself. So this isn't something I've, you know, that particular at that particular piece of data. I think, gosh, do we have that anywhere? Do I know what that is? I don't think I do in our overall system priorities as a kind of, you know… As a Commissioner we identify the working age, I'm going off the top of my head here.. Working age population, the areas with the greatest impact on health they are…. So there's anxiety and depression and mental health and then probably the other really key areas are an MSK type condition, some of those may have been brought on so that this group might be a kind of subset of it. But we don't collect, as far as I know, I don't think we kinda routinely collect our kind of population data on injuries and return to work data. You know that would be a great statistic to have, wouldn't it? (COM03)* |
| **3e) Tension for change**  The current situation is intolerable and needs to change. | **Buy In** | *But what we have seen is a real opportunity and so actually government bodies are DWP, NHS, England Department, Health and social care reaching out to the professional bodies, but in particular reaching out, reaching out to out of the AHP bodies, to occupational therapy, in recognition that actually we need to take a new approach, a fresh approach. So, there's been two areas of focus. One has been occupational health. And just the recognition that occupational health service are varied and most people are employed in small to medium sized enterprises and therefore there isn't an occupational health department or you know, it may be they haven't even got a contract with a with an occupational health provider. And when they do access occupational health, it tends to be an assessment over the phone and some recommendations in a report job done. So that's one area. (COM07)* | *I think the other barrier is that all monies and focus and public attention goes into acute hospital care, so if you ask a member of the public what does the NHS mean for you, they think of their GP and A&E unless they are having direct input from services, that is that. That's the focus and that's where the money is going into, you know. You know, keeping hospitals afloat, you know? And that's what sort of catches the headlines. So all the policies are in one direction, the funding is going in the other direction and public awareness sits in the other direction. So I think one of the huge barriers is actually public perception. (COM07)* |
|  | **Employer Engagement** | *I don’t feel as though I’ve got the support in place that’s required to deal with a complex situation like [employee] has got. It’s not only physical, it’s mental health issues as well, and anxiety and I want to do a great job (EMP03)* | *It’s that fear of disability discrimination that drives that, because the employer’s wanting to tick a box to say, “Right, well, we’ve done everything that we can, you know, we’ve considered adjustments, but there’s nothing reasonable that we can do in this situation,” so there’s a bit of ass covering. (EMP04)* |
| **3f) Compatibility**  The innovation fits with workflows, systems, and processes. | **Buy In** | *So it's all about all about community services, integrated care, improving flow and then also how we developed joined up community services. So that kind of integrated holistic, more proactive preventative care (COM03)* | *I think trauma orthopaedics, if you tried to say to them, “Do you need it?” they’d probably go, “Well, no,” because they’re in and out of hospital. If you said to the GPs— And that’s the other thing. Do they sit in the GP surgeries? So you don’t look at it as a secondary care service. It’s more of a primary care service because there could more of a larger role there about an employment specialist that looks at all the fit notes and all of that kind of stuff within a GP surgery. But you would not want to open the floodgates with an OT doing that because nobody would want that job (TI07)* |
| **3g) Relative Priority**  Implementing and delivering the innovation is important compared to other initiatives. | **Buy In** | *I think we're looking across all kind of long term illnesses and how we get people back.(COM02)*  *The long term aim here is that there's lots of evidence to show you that if people stay out of work, they're far more at risk of mental health problems, obesity, you know, reduced physical activity and all this, everything that comes with that. So and that then causes people to take up multiple appointments, disease monitoring,. all of this that comes with it. So actually, yeah, if you can, if you can reduce all of that from happening, then you're going to reduce GP appointment requests, monitoring of conditions that they won't develop because they're fitter, more active, you know, all of these other things, which.. and that will reduce GP workload, won't it? (GP03)* | *The financial constraints you know, in terms of the cost of onward rehab, it is the biggest thing on a day-to-day basis. And then in the background I would say is the fact that basically there's so many competing priorities within NHS England and or ICBs, it's the biggest challenge is keeping rehab on that agenda at all. (COM04)* |
|  | **Patient Needs and Outcomes** | *I suppose it was like – it was like the unknown for me really, and I wouldn’t have known what to do. I’ve gone through a series of things, and I don't know if you’ve seen all the emails me and [my occupational therapist] have shared back and forth really, actually – if I hadn’t had that, I would have been either to the GP or the hospital or trying to get some advice. (PA14)* | *Two of them, I managed to make contact with initially and they shared with me their mental health history and some of their stresses and anxieties around money and jobs and work and housing. So I think actually in those two cases their lives were quite chaotic and stressful to begin with, so I don’t think they have the capacity to engage with this. (TA01)* |
| **3h) Incentive systems**  Tangible and/or intangible incentives and rewards support implementation and delivery of the innovation. | **Patient Needs and Outcomes** | *They also provide private healthcare for us as well, which I opted into, and so things like my arm still being broken I was able to see a consultant a little bit quicker than I otherwise would. (PA12)* | *So one patient had got a legal claim going on which actually many patients do have, but for this one there just seemed to be the possibility that the legal claim or the things that were happening in terms of the legal and insurance side would get in the way of this patient returning to work more quickly, or get in the way of the patient making decisions about whether to return to work or not. (TA04* |
| **3i) Mission alignment**  Implementing and delivering the innovation is in line with the overarching commitment, purpose, or goals in the Inner Setting. | **Employer Engagement** | *So we have this approach of compassion and caring, because that’s what our employees tell us they expect from us as an employer. So, definitely we are aligned with that mission (EMP01)* | *Would you just think, oh come on, pull your socks up, type attitude to it? Or we’re a business, we’re paying you good money to do that and you’re not performing. There’s plenty of people we’ve got in this business who don’t perform. (EMP03)* |
|  | **Buy In** | *I also think there's again the prevention here is very much about secondary prevention and if not tertiary prevention in terms of what would what we're doing there. So it and that's secondary prevention is very much a priority of what we want to be doing across the across the ICB at the moment, in terms of that and often sometimes harder when we've got the pressures with the resource pressures that we're seeing in terms of the here and now there. So I think it is from a from a clinical point of view it's very much something we're looking at in terms of prevention there and getting ahead of the curve in the situations where we've had the major injury there. (COM02)* | *So we know that you know, global population health people are living for longer with health conditions, multiple health conditions and in the UK, we're seeing a real rise in that. And though people are living for longer still, that's still going on the out, not as much as it has been, but it's still on the up. But what we're finding is healthy working life is not matching length of life, so it is an area of concern (COM07)* |
|  | **Patient Needs and Outcomes** | *I’m due to retire in May, I’m 61, I’ve done my bit and stuff, but actually I didn’t want to – I wanted to get back to work because I didn’t want to sort of feel – and have to be retired on disability grounds and stuff, so it was important to me to get back to normal and then retire on my own circumstances, rather than it be forced upon me. So that has really helped me to get there really. (PA14)* | *They’d even tried to get me more sick pay, but they couldn’t because it was controlled by the head office and all the systems so they couldn’t get me any more than got, unfortunately. (PI03)* |
| **3j) Available resources**  Resources are available to implement and deliver the innovation. | **Buy In** | *Nothing seems to happen very quickly on the NHS. So that’s frustrating for everybody, because the employer’s thinking, well, what are you doing to chase it up? Are you phoning your GP? Are you phoning your consultant? Have you phoned the secretary’s office? And the employee’s feeling defensive thinking, I’m doing everything I can, I don't know where it’s at. The fact that there’s a proactive service that can get them back to work sooner I think is a real key selling point here, for everybody involved. (EMP04)* | *I suppose I'd say we don't commission anything specific for that group. And therefore, I would imagine that the that clinical staff have to try and find the resources necessary for that kind of group of patients. (COM03)*  *There's a lot of generic working having to be done to ensure that the wider NHS and social care system just ticks along. So actually if you start to pull people out of that and say you've got the expertise to deliver on hit on this, what happens to those other services? (COM07)* |
| **3k) Access to Knowledge**  Guidance and/or training is accessible to implement and deliver the innovation. | **Mentoring** | *I had one that was quite challenging, and I called [Mentor name] a few times about that one to kind of get the ad-hoc kind of support. And the other ones were bit more kind of probably just supporting just as a team, kind of discuss cases and gets a bit more support with the structure and what the expectations are and just discussing different topics, that’s kind of just to help our experience and learning. (TA02)* | *I think just finding a kind of slot that works for multiple clinical psychologists when this is work that they're sort of fitting around. So, I think that's helped where we have now a couple of clinical psychologist mentors and we have sort of different slots that we kind of offer on different days and different times of the week. Even then, I think the sort of availability aspect can be a bit tricky, but it's just about getting kind of 4-5 fairly busy people who have, you know, doing this in a part time capacity together at the same time. (ME03)* |
|  | **Employer Engagement** | *Because what I find is that the employers that have this situation are like, “Oh my God, so and so’s just suffered this car accident,” they contact HR, they contact employment lawyers. “Okay, what do we have to do?” And that’s when they do start to get defensive, because they’re out of their depth, they don’t know what they’re doing. So being able to say, “Right, here’s your signposting for the first things, here’s the next best step for you,” so that they feel supported as well as the employee. (EMP04)* | *Sometimes the clinician expects too much from the employer in terms of reasonable adjustments, when it is affecting their business or the business relationships. I mean, the pathways sounds absolutely fantastic, but in practice, because there’s so many people involved in it, there could very quickly arise as I say communication breakdown or just – or even people going on leave or on promotion, leaving gaps behind them that don’t get filled. (EMP02)* |
| 1. **Individuals** | | ***The roles and characteristics of individuals*** | |
| ***Document the roles applicable to the project and their location in the inner or outer setting [Outer: Commissioners, Clinical, Legal Inner: Mentors, Therapists, Patients]*** | | | |
| **ROLES Document the roles applicable to the project and their location in the Inner or Outer Setting.** | | | |
| **4a) High Level Leaders**  Individuals with a high level of authority, including key decision-makers, executive leaders, or directors. | **Buy In** | *To do things differently at scale and with impact, you have to change your thinking 'cause. If you don't change your thinking, you won't change behaviour. And I think that that's where somehow an organisation has got to take the metaphorical time out to think differently in order to do differently. Otherwise, you just keep on doing the same thing. And I think that's where the ICB at a board level is really meant what it's meant to help deliver to try and create space where people can think differently. (COM06)* | *I think specialised commissioning is potentially being delegated to ICB or to our ICB being the next year. We're currently working through what that really means for us as a ICB, given that it has been both nationally and regionally done up until now, what that means for us as an organization and as a system where we have that responsibility and accountability for that. But how would then work with our other colleagues across other ICBs and our region? And so I think that's a priority to make sure we get it right. We get the right people involved with that, we have the right conversations. So we have the right understanding so that we can then look at what services we need as a system and what we need to put in place there. So I do think that's going to be challenging, (COM02)* |
| **4b) Mid Level Leaders**  Individuals with a moderate level of authority, including leaders supervised by a high-level leader and who supervise others. | **Employer Engagement** | *So any quality research I believe would need to actually not just headline the research, but actually convert it into an HR policy, which is in effect – almost like the Ikea thing, it’s on the shelf, it’s ready to go. (EMP02)* | *If I went to speak to a transport department team, they’re almost all men, if I went to speak to the education or health sections of the organisation, they’re almost all women. I tend to understand that almost every team or role has its own personality. And unless you're making some effort to reach that, or breach it, even better, then you're going to rub up against the idea that they know their role, and you don’t understand them and then they’re bunkered…. Their buy-in is really important, because if they don’t recommend it comes out of their budget it won't happen. (EMP02)* |
| **4c) Opinion Leaders**  Individuals with informal influence on the attitudes and behaviors of others. | **Buy In** | *I run a network called the [EMP02], and it builds up on around 20 years’ experience in working in policy areas in the disability movement, mainly for large charities… we have the expertise within our ranks, but we’re an organisation that’s really concerned with very high level policy interventions, so at the moment, we’re trying to see the Chancellor. I’m a mixture of the advocate and the policy – and a lobbyist basically, and of course I’m interested in persuading employers, particularly because they’ve got skill shortages, many of them, so this should be a really great time to talk to them. (EMP02)* | *And in a way, a lot of the issues of disability fall into the four quadrants, and so people don’t learn things so much, because they don’t know how long it takes to learn something, and they don’t know what the value of what they’ll be learning is. (EMP02)* |
| **4d) Implementation facilitators**  Individuals with subject matter expertise who assist, coach, or support implementation. | **Mentoring** | *And we spent most of the time talking about participants, talking about the intervention, talking about complexities of cases and what to do, making suggestions. Me encouraging the other OTs to say what they think as well, so it’s not all coming from me. And the more we do it, the longer we do it, then the more confident they are at coming forward and providing suggestions themselves. So for me, I kind of see that as a reflection of them feeling more expert in delivering the intervention, or more confident (ME02)* | *And every time we find something out that we’re slightly worried about, we want to try and take action on it, in the study, and then that creates more work, and it makes it more difficult for me to balance my duties. (ME02)* |
| **4e) Implementation leads**  Individuals who lead efforts to implement the innovation. | **Mentoring** | *[Trial leaders] and the administrative team have all been really responsive with those queries. So that's been helpful. I wonder, I mean. And I feel like if I'd have said, actually I do need you know more training around, mentorship. And so, if I didn't have previous experience of mentoring and supervision. You know, I think as with everything else, whenever we've identified a learning need, the team have sort of tried to direct us (ME03)* | *I would have definitely valued a bit more notice and communication about when the trainings were going to be. I think we ended up getting the dates quite late on and, you know, I worked around it, but I think I guess the future setting up and getting therapists involved, it’s good to have a bit more time and planning in it. (TA04)* |
| **4f) Implementation team members**  Individuals who collaborate with and support the Implementation Leads to implement the innovation | **Mentoring** | *I think my role as a mentor is to give the OTs confidence that they can do it, and reassurance that even though it’s – because I think the fact it’s a research project freaks them out, so it’s like saying, “Actually, the research stuff is just like a clinical process. You just have to follow it. The treatment is just like being an OT. You know, we’re giving you the guidelines. Do it.” (ME01)* | *[It would be helpful] if the OTs understand what the recruiters’ roles were, because I don’t think recruiters necessarily understand the intervention either, and I think one of the big things is getting the OTs, for me, it’s getting the OTs to talk to the recruiters, and also giving the recruiters feedback about how the patients are. Because otherwise it’s just thankless. “Oh, would you like to be part of this study?” “Yeah. No. Okay, next one.” (ME01)* |
| **4g) Other support**  Individuals who support the Implementation Leads and/or Implementation Team Members to implement the innovation. | **Mentoring** | *I see the people that I’m providing mentorship to are very experienced clinicians themselves, so I think it’s more like peer supervision than clinical supervision for someone who’s training more. But I suppose because I’ve been involved, I think it’s probably my involvement in ROWTATE to date that’s meant I can help them with more of the practical elements of being involved in the trial and providing the intervention, and having done it previously myself as a floating therapist. Because I’m only dealing with floating therapists as well. But I would say the rest of the mentorship is more along the lines of peer supervision, which I would regularly involve myself with. (ME04)* | *I do think for future studies you need something, like because it’s up to them whether they come or not, but if they don’t turn up then how do you know they’re doing it? And is there any recourse? Do you know what I mean? Because in [PREVIOUS STUDY] we had somebody who did not turn up despite the mentor trying really hard to make contact, and she was going under and she didn’t see the patients and the patients were left. So I think there needs to be some mechanism of even if you don’t come to mentoring, we need some method of knowing that you are delivering what we are asking you to deliver. (ME02)* |
|  | **Buy-In** | *What we have seen is a real opportunity and so actually government bodies are DWP, NHS, England Department, Health and social care reaching out to the professional bodies, but in particular reaching out, reaching out to out of the AHP bodies, to occupational therapy, in recognition that actually we need to take a new approach, a fresh approach (COM07)* | *People have a very limited perception of occupational health and I would say generally not a positive one. It is seen as almost a performance management exercise rather than, you know, an assessment and intervention on your behalf. So, I think we've got all those barriers to to overcome. (COM07)* |
| **4h) Innovation deliverers**  Individuals who are directly or indirectly delivering the innovation. | **Patient Needs and Outcomes** | *The support that I’ve received from OT has been amazing; amazing from giving me, how to understand, giving me targets to work against and almost like psychological support as well. (PA02)* | *I think it varies quite a lot in terms of …. what you've got at the moment is a very fragmented service. And you know, if you said to me, are people who are out of work or can people who are out of work get access to psychological support? The answer to that is yes. But you know, the wait for that psychological support is very long, the wait for physical support and rehabilitation in terms of physiotherapy, it's probably much shorter, but there's still a wait. So, I suppose the elements of it, some of the elements of it are there and available, but it's not joined up (GP05)* |
|  | **Patient Needs and Outcomes** | *I’m self-employed anyway so I sort of fit it in around my current work and I had some good space anyway, so it hasn’t been too much of a problem for me. (TA02)* | *The time it took to constantly be calling them or setting aside time to arrange an assessment with them and them not being available. Or setting aside time to do the assessment and being interrupted by someone else on the hospital ward. Or just not contacting me. And telling me that everything was going to be really difficult but then disengaging. That was just very time consuming. (TA01)* |
|  | **Buy In** | *I do this alongside full-time working, so that’s why my numbers haven’t been as high as perhaps other therapists. But I still feel, even though I’ve seen eight people over the last year, that I have a sense of the process and what it’s like delivering the intervention. (TA06)* | *If you were only, in theory, doing it on a morning per week but you’ve got employers, or even sometimes when it’s not being someone disorganised but the employer of that participant isn’t available on the Tuesday morning that I’d said I was doing ROWTATE but they are available on a Friday morning or on a Wednesday but I work on neuro rehab at those times, it’s— I worked it out, I did flex and do those things, but I think the biggest problem in some way came from the expectation that you could deliver it like we’d been told in that half day. (TA05)* |
|  | **Remote** | *From our side it’s really convenient to work from home. It’s really helpful. I can fit people in a bit more easily because I do visits myself so I can kind of slot people in when I’m back there for an hour, I can fit them in there. So actually I’m seeing more patients or participants than I would be if it wasn’t virtual. (TA02)* | *I guess this is why I might have been doubting whether I was doing things right because, if that is the case that a lot of people are more than happy just to deliver it remotely but I was finding that it didn’t work (laughs), it’s like, well, it must be me then, it must be me. (TA05)* |
| **4i) Innovation recipients**  Individuals who are directly or indirectly receiving the innovation. | **Patient Needs and Outcomes** | *I was getting really, really angry with her. “Don’t get angry with me. I was like, “I have every right to be angry with you.” And then when ROWTATE finally called, I think it was middle week, Wednesday, she realised no, you can’t work. And then she realised and she put everything away and stopped. (CAR01)* | *You’re leaving it to a guy who has mental health issues from before anyway, for something else. He was struggling with anxieties, stress, you name it and then you’re expecting him to be coordinating point for his own wellbeing and how on earth do you do that? (EMP03)* |
|  | **Employer Engagement** | *I’d be really lost as a line manager, to know what the hell is going on with him, I really would do, because it’s just not clear. (EMP03)* | *I’ve put into our occupational health team, so a company called [OH Company A] I think you probably saw the reports. When I had the conversations with him, it’s so then what is the next steps? He’s like, I’m waiting for someone to do an appointment for X, Y, Z and then he’s waiting for that appointment to come along, while seeing someone else (EMP03)* |
| **CHARACTERISTICS** | **Document the characteristics applicable to the roles in the project based on the COM-B system or role-specific theories** | | |
| 1. **Need**   The individual(s) has deficits related to survival, well-being, or personal fulfillment, which will be addressed by implementation and/or delivery of the innovation. | **Patient Needs and Outcomes** | *I didn’t feel suicidal, but I did feel very depressed sometimes and very lost and ROWTATE stopped me. It gave me some signposts and a gentle, regular steadiness to find a way of moving forward. (PA07)* | *Understanding that obviously there's a cost, there's a wider cost to this on understanding the impact and that's where I think kind of working with the kind of wider teams, local authority really understanding those wider, why there are aspects that economical growth for the for the system, which is part of our mission as an ICB, is to kind of improve that economic growth. (COM02)* |
|  | **MDT** | *I guess this is that we want to get people back to work back to functioning, because then that I guess it is.. I guess it is preventative medicine because that does have a knock on effect on their mental health and then when their mental health is better, they will use services less, you know, and have, you know, and then also adding to the economy and all this, that, that and the other. So it is absolutely worth it, isn't it? And should in theory also help where, then again it depends I guess what their injury is, but if people are sedentary and not really doing much, then their pain's going to be worse. All of these things, whereas if they become active, you know they're active, they get back to go to work, then all of that is going to have a positive effect on their physical and their mental health, isn't it? So I think trying to, like with commissioning services, selling it as a preventative thing rather like, you know, we're trying to be proactive, aren't we, rather than reactive to things. That is a good way to do it. (GP02)* | *I can think of one example where there's a lady who had an accident on her horse, which resulted in some spinal damage. And she has become, unfortunately, addicted to painkillers and benzodiazepines, and she's morphed into a bit more of an addiction problem. And she's not worked, and I suspect never will. (GP04)* |
|  | **Employer Engagement** | *You’d want to know enough information to make the right decisions for the good and benefit of that individual and the company as well. You’ve got to protect the company’s interest as well in this (EMP03)* | *Some people they want to please their employer, they want to please their line manager (EMP06)* |
|  | **Mentoring** | *So there was a lot of explaining of those things in more detail than we could ever have hoped to cover in the training sessions. This is how different people within an employing organisation talk to each other, this is what they do, these are their responsibilities. So we talked about – did a lot of talking about, what does the finance director do, what does the CEO do? So that they could have a better understanding of this unit. Because they’re used to working with families, and they would understand what a spouse does, and what a child does, and maybe what other people sort of further away in the family unit, what they do, and their responsibilities, so it’s about translating that into an employer organism, if you like. (ME02)* | *She hadn’t mentored anybody, so she was having – it was like, “Oh my God, this is the...” she wasn’t panicking, don’t get me wrong, but I was thinking, “Yeah, this is normal. This is what we’ve had before,” do you know what I mean? And it was like we just have to deal with it, you know, this is part of the process and it gets easier. (ME01)* |
| **Capability**  The individual(s) has interpersonal competence, knowledge, and skills to fulfill the role. | **Employer Engagement** | *I see things in a different way that I don’t feel as though I’ve got the support in place that’s required to deal with a complex situation like [employee] has got. It’s not only physical, it’s mental health issues as well, and anxiety and I want to do a great job (EMP03)* | *Thankfully, in my 20 year career, this is the first time I’ve ever dealt with a severe injury like this. So, I take the assumption and I may be wrong, that the prevalence of these sort of injuries is low, but of course the impact of them is going to be huge (EMP01)* |
|  | **Mentoring** | *Having previous experience I think has helped me (ME02)* | *I think they all did, they were all good occupational therapists. So they had good skills as occupational therapists, what they needed was reassurance and confidence building, that those skills are transferable into other specialities and into vocational rehab really. (ME04)* |
|  | **Patient Needs and Outcomes** | *I was always fit and active before the accident anyway, so I sort of had an idea of what kind of things I could be doing to try and get some strength back in, because first of all my legs as well as my arms were very weak, and we were just walking to try and just get my strength back really. But if I hadn’t had that kind of knowledge, I think because it was so delayed, I don’t think I would be where I am now. (PI03)* | *All I can remember is there was somebody came round and said, would you like to do a survey? And I must admit, I probably didn’t take it all in. But I am glad that I signed up to it now. (PA05)* |
|  | **Remote** | *I’ve got one participant who’s in her 60s and she has no problem with using MS Teams at all (TA06)* | *I think the younger generation will be able to cope with it better, having a video call, because they know more about these mobiles phones and computers than what I do. I mean, you know, sort of, because when I grew up there was no computers really. (PA01)* |
| **Opportunity**  The individual(s) has availability, scope, and power to fulfill the Role. | **Mentoring** | *It definitely had me develop as an OT, and lots of transferable skills really as well. (TA13)* | *In a way, my private business needs to come first. And then ROWTATE comes second. So if I’ve got time and I know that I can pick up another participant, then that’s fine. (TI07)* |
|  | **Employer Engagement** | *The fact that they could be taking more proactive option to make that person feel better sooner, and to get them back to work, you know, in a functioning capacity, I think would be attractive to employers. (EMP04)* | *From an employer’s perspective, so let’s face facts, people are running a business to sell their products and services and to make money and to crack on with that, and to have someone who’s key to their business out of the loop for whatever period of time, indefinitely, is a challenge to business owners, no matter how much they love the employee, is it still a challenge, they’ve got a hole there that they need to fill (EMP04)* |
| **Motivation**  The individual(s) is committed to fulfilling the Role. | **Patient Needs and Outcomes** | *[The OT] really given me structure, given me really good targets and goals to work towards which consequently I’ve made excellent progress and the motivation that she’s given me and the emotional support has also, you know, given me the drive to take some control as well of my recovery and start working on targets with that, I’m not just waiting for targets to be set, but actually thinking, okay so, well maybe I can do a bit more and maybe I’ll do this and keep working on expanding the targets that have already been set, when I’m able to, if that makes sense. (PA02)* | *I mean, there’s every possibility I would have done it myself.  But I don't know.  And again, it comes down to structure essentially, it’s not just a, you know, everything down to me, my responsibility, there’s a plan in place essentially. (PA06)*  *I’m basically a very lazy person – well, I say I’m basically a lazy person, I work hard, but I relax too much when I’m not working, I do struggle. So I find the impetus to do things properly – I can do them, but I just struggle with that. (PA13)*  *Yeah, the element that the pain of the injury, the pain, the injuries, subsequent surgeries, for example, or life context can get in the way of that motivation. (TA14)* |
|  | **Remote** | *I suppose that in the last maybe year or two, and I would imagine that my experiences are perhaps a little bit similar to others, is that we're seeing a slight, I don't know, migration or slight change in the reality of I guess things like virtual consultations. So, for instance, patients requesting a sick note via a form that they're filling in, rather than necessarily an in-person appointment, or even telephone necessarily interaction. So, I guess that's probably where I'd see things having changed a little bit. (GP01)* | *I think it’s a lot easier to cancel someone who you’ve never met, who you just speak to over a screen as opposed to cancelling someone who turns up at your house every other week (TA03)* |
|  | **Buy In** | *So we recently like had, was looking at like an exercise programme in elderly people to try and prevent frailty, so to try and prevent them becoming more frail and hospital admissions this, that, that and the other. So that would that's the sort of thing that has been commissioned recently. I guess the aim is to move from treatment and medicines to preventative measures, so to try and you know, stop things from happening before they have happened. (GP02)* | *So now basically you get telephone consultation, and you know, it is your exercises, so that's that. And then other physios are not going to be happy just doing that because that's not what they joined up to do. (COM06)* |
| 1. ***Implementation Process*** | | ***Implementation Process: The activities and strategies used to implement the innovation.*** | |
| ***Project Implementation Process: [Document the implementation process framework and/or activities and strategies being used to implement the innovation. Distinguish the implementation process used to implement the innovation (activities that end after implementation is complete) from the innovation (the “thing” that continues when implementation is complete).]*** | | | |
| **5a) Teaming**  Join together, intentionally coordinating and collaborating on interdependent tasks, to implement the innovation. | **MDT** | *I'm heartened by the option of referring into a psychologist because I do think with my previous experience and background, I often worked with people following major trauma once they had been through the physical health services. And they would work through their programme. And then if they couldn't just then pick themselves up and continue with their life merrily, because it was often after rehab that the psychological impact would hit people, it would then be left to get so bad that they ended up coming to secondary mental health services and I'd have to sort of, you know, would be picking up again. So, I think they, I think the psychological aspect is really important. (COM07)*  *And seeing how you can mesh together as a team of two, to deliver the intervention, for the benefit of the patient, participant. So that’s where it’s worked really well, and I’ve had some stories both from clinical psychologists and from OTs demonstrating great joint working, yeah.(ME02)*  *I think that combination of sort of OT and psychology support where those needs are about kind of returning to work or education. I think that, you know, they would say again from feedback that I'm familiar with that that's you know been a really useful combination in terms of addressing, you know, what are their main goals at that time? Some of that's about, you know, understanding how the injury is affecting them, what that means for them functionally. You know what's kind of what's their capacity for returning to work, how are they going to kind of plan and do that in a graded way in a way that's kind of sustainable. But also, there's a lot around that kind of, you know, meaning making sense of purpose, work life balance. I think the sort of psychologist role is really kind of a helpful affordance to have at that time as well. (ME03)* | *One of the things I felt that the pathways were all good, but they relied upon a lot of different people with expertise interacting with one another. And that tends to break down very quickly, because individual silos, communication issues, even people not getting on with each other kind of thing or accept somebody else’s authority or judgement. (EMP02)* |
|  | **Remote** | *The OTs that I work with, sometimes they'll sort of ask “Oh, do you know anything about this person?” So, they picked up this referral. “They're linked to a clinical psychologist, but I haven't heard back from them”… even if it's sort of virtual on teams and even if it's a kind of rolling workforce or people kind of joining and dropping out. So, what can you put in place to sort of structure and encourage that, you know, those points of connection over time. (ME03)* | *I think the ones that were trained together, they’ve definitely – I’m sure that they’ve got a better connection with each other and feel able to reach out. Some of the OTs that have come in later on, and they’ve not seen [CP6], [TA11] or, you know, any of the other clinical psychologists at all, so there’s just naturally a little bit more distance between them. Professionally they’ll reach out anyway, but maybe there’s a slight hesitance at reaching out, or the dynamic is slightly different, more distant in some way, than those that were trained together. (ME02)* |
|  | **Mentoring** | *I find the supervision process really helpful that we have every month... I really valued the support… It’s really nice to be working as part of a team and I think here .. I definitely feel like part of the ROWTATE team, which is nice and really supportive so if any of us are having any issues then we’ve got each other to kind of bounce off and give support, which seems really good. (TA03)* | *I think having the mentoring together occasionally, I think that would be really helpful because there were a lot of issues about how do I use the psychologist? The psychologists were very good at setting boundaries, but that meant the OTs were left to pick up everything and I just think sometimes having a session, you know, where the OTs and the psychologists could discuss their patients [would be good] (ME02)* |
| **5b) Assessing needs** | **Collect information about priorities, preferences, and needs of people.** | | |
| **Innovation Deliverers**    Collect information about the priorities, preferences, and needs of deliverers to guide implementation and delivery of the innovation. | **Mentoring** | *The skills that you use to support somebody in that way, it is confidence because you've got to have the professional authority to manage challenging conversations and to be speaking in sectors that many occupational therapists don't normally speak in. So, you know, OTs will be used to working with families, speaking to families, speaking to domiciliary agencies, etcetera. Commissioners, or going into a business environment and being able to speak in a commercial business environment. So it's just it's confidence around talking to different audiences and it's confidence in applying those transferable skills into an unfamiliar setting an area. (COM07)*  *The mentor hasn’t got the answers; we’ve all got the answers, and to make it a sort of discussion, a safe place to say whatever. And I feel confident enough, and TA10 did, what was really good about TA10 and also that I feel, the more experience you get, the more confident to say you don’t know. And when you knew and you’re trying to prove to yourself or think I should know the answers, you don’t say it. Do you know what I mean? So I think, you know, I think it was alright for me to say, “Well, actually I don’t know, but thinking about it, let’s think what could we do,” do you know what I mean? I think just showing that there isn’t always answers is helpful. (ME01)*  *I do think they did all have the relevant skills, because they were all occupational therapists. But they needed some guidance to be able to feel comfortable in a different role but use the same skills. (ME04)* | *I think they’d have to have specialist VR knowledge because I think it is the – in my personal experience, it is the very specific questions about employment law that worry clinicians, do you know what I mean? And I think it’s also about having that space to have mutual support, so I think if you could have a meeting every two or three months, two months, and a named person to go to or a named email that everybody could input, that would help. (ME01)*  *I think practically, if you’re going to be a mentor on a study, you need to be involved in the training from the beginning. But if you’re a mentor called in halfway through, you need to go on the next training session or have a session for the mentor to say, “Look, this is what we taught them.” … the learning for future studies is getting mentors to be part of the training programme so they know what they have been taught. (ME01)*  *I always have imposter syndrome, I’m very good at having imposter syndrome, (laughs) so I never feel like I’m the right person for any role I think. But as it goes on – as it’s gone on, I think I’ve gained confidence in that. Certainly, I think I had the elements of the delivery of the study that I felt confident enough to share, having done it myself, and actually having some active participants in the trial as well. (ME04)* |
|  | **MDT** | *So along the way we developed clear guidance on a pathway for psychology intervention, particularly I think in the early days there were some issues around safeguarding responsibilities, when someone was raising safeguarding concerns, suicidal ideation and things like that, and being much more clear about who was responsible for dealing with that. And then also guidance on – creating more guidance for the psychologists on how quickly they needed to respond and what was to be expected of them, so yeah, things like that, that have developed as it went on, based on the feedback we were getting from the OTs. (ME04)* | *I could say as a floating psychologist, I don’t feel like I’m under pressure to take on more hours than I signed up for, but I know some of the OTs in [AREA] are really feeling like they can’t keep up, or that they’ve got to work many more hours than they intended to or signed up to. So I guess it’s kind of a bit of an issue of consent there so you’re kind of consenting to so many hours and actually the workload far exceeds that, particularly for the OTs. But it would for the psychologists without that option of the floating psychologist. (TA04)* |
| **Recipients**  Collect information about the priorities, preferences, and needs of recipients to guide implementation and delivery of the innovation. | **Buy In** | *I think one needs to understand the, the links across a local authority and there because that's where ultimately whether it's education or whether it's, well it's employment that's ultimately what we were hoping for you - we're using ROWTATE to enable people to get back into employment or education so then progress so that their lives so I think there's something about that there is aspiring to, what you're aspiring to, for those individuals, understanding what that means and what success is for the individual and what successes for the organizations that are involved. The how, clearly it depends. Someone could come in with a significant major injury but actually recover quite quickly. But someone who can come in with a following, a stroke or something that significant stroke and actually take many months to even recover to a point where we can start to think about this discharge there and what that means. So I think understanding the kind of range of different conditions will have an effect in terms of the success. Well, the speed of the speed at which we deliver this as well. But that may not always mean success. So success will be different for individual. Some success will be actually just getting back into some sort of education. Others will be about and, and that may be very basic. It may be about going back into a full time employment, which may never be a possibility for some as well. (COM02)* | *So the barriers, one is that we don’t understand. As I said before, we haven't ever – I don’t think we’ve looked at it well enough to understand whether there is a need around that population cohort. (COM01])* |
|  | **Patient Needs and outcomes** | *You guys really, really supported in hammering into her, you know, “You’ve had a brain injury. You need time to recover and, you know, stressing yourself out, you need to switch off and move away,” and she did. (CAR01)*  *They’ve contacted me to ask me if I’m okay and if I’ve had any problems with, do you know, sort of nightmares or flashbacks and things like that, any physical problems. I went back to work after seven weeks and yeah just carried on as normal (PA01)* | *I would consider referring these people to our social prescribers because they look at, you know, a holistic view of everything. So they also would help them like financially, with benefits all this sort of thing and then they can access them a little bit more quickly. For more, so that would be looking more at the kind of I guess, not mental health, although they can offer some mental health support but not, you know, not really trained stuff, but looking at other things like actually helping them with benefits, getting them back to work, helping them with those sorts of things. So social prescribers might be helpful in this scenario. (GP02)* |
|  | **Employer Engagement** | *Almost like they appreciate the certainty of, “Actually, this person’s not going to be able to work for 12 or 18 months, and let them focus on their rehabilitation privately, and as an employer, we appreciate that you’ve ticked your box, but we’ll be back in touch with you when they’re able to return.” Rather than this kind of keeping them hanging on a thread in terms of three months, six months, nine months, 12 months, oh, they’re coming back soon, you know, when actually the reality is that they’re probably not, they’re not ready yet. (EMP04)* | *there’s no one person who is saying right, you are now waiting for this appointment. You are now waiting for that result, MRI scan, drugs. He’s been bounced from doctor to doctor with different drugs, which we could discuss later, about not great in a safety critical environment where we work in sometimes and things like that. So, yeah, for me it is about coordination. (EMP03)* |
| **5c) Assessing Context**  Collect information to identify and appraise barriers and facilitators to implementation and delivery of the innovation. | **Buy In** | *the second area was recognition that rehabilitation is part of supporting people to return to work on and remain in work. And so that's how we you know, started to become focused, colleagues that you know are experts in this field who are members of the college talk about vocational rehabilitation being an approach, and then you've got different mediums for delivering that vocational rehabilitation. So we've adopted that, that language, that approach and are using that in our strategic conversations. And that's really well received, people get it, they understand it. And so, what they're looking at is working with, and we've got 4 government so it's always a slightly different approach with each government. We're looking at the initiatives that they're driving forward, but then saying actually where is it and we'll talk about the infrastructure or the ecosystem around coaches, work well initiatives, where is the ecosystem around that, those individuals? Because they will fail if they don't have the mentoring, the support and expertise, and to be able to refer in and refer, you know, and for people to pass out. So, what we know is traditional services, you know people get referred to, they get so many sessions, sometimes it's quite fixed how many weeks that they get or how many sessions they get. That person's then discharged and off they go. But we know with long term health conditions, people can manage their health and their situation very well for periods of time and then there will be periods where they need a bit more support and a bit more help. And so we're also talking to different civil servant groups thinking about how can we create access points. And how can you know if somebody's been on the books, is known to the service? How can they dip in and dive out and get different levels of support rather than the traditional fixed packages that have happened before? (COM07)* | *I think it’s easier for the very big or the very small, but the problem is in the middle ground, where obviously large numbers of employers sit. (EMP02)*  *It could as long as it’s converted as I said into an HR policy, and preferably have government support to it to send this round as a suggestion – even the suggestion that this should be part of the kind of the whole manual in effect of policies. This needs to be pitched at different levels I’ve suggested, the very small businesses, less than 10, the businesses that are 10 to 49, and then the businesses that are 50 plus. The easiest ones to liaise with tend to be always the ones that are actually 250 plus, which there’s 6,000 businesses in the UK of that size. But they’re the easiest ones to deal with because they’ve got HR departments and they’ve got people with specialist knowledge, and they’re big enough to have seen most things. But because they’re the easiest to liaise with, all the experiences tend to be on that, whereas it’s the middle ground I think is the most important. (EMP02)* |
|  | **Patient Needs and Outcomes** | *I think it’s been very helpful because I was in hospital out of my local area, so when I came home I was sort of, didn’t really have a great deal of contact with anyone, so having that contact has, I’ve had like that regular contact every couple of weeks in the early days, so it was like somebody to talk through things and make, reassure me that everything was, you know, what I was going through was okay as well. So yeah it’s been useful. (PA03)* | *I think given how determined I was at the time to go back to work there was just nothing stopping me. I think if someone had the conversation with me now that would be a very different conversation, but at the time I was just like, yeah I’m fed up of being at home, and I had exactly the same feeling, like what, like within the week being discharged from hospital from my recent surgery, I was like, I’m fed up, I’m bored, let’s go back to work, and I was like, hang on a second, I’ve just gone through all of this why, I know this is wrong, and yet I still had the same urges to just be normal again. So I know that that’s probably a personal drawback but it might be something that everybody experiences because they don’t want to be ill, they don’t want to be unwell. (PA12)* |
|  | **Remote** | *Because there’s no physical assessment, so there’s actually no value in physically being present. It’s more convenient, it’s more cost-effective and yeah there’s no, there’s no barriers from my perception. (PA02)* | *I don’t necessarily know that there is a solution to it, and it might very well just be something that I faced or just people that have all these phone calls and appointments and everything going on all at once. (PA04)* |
| **5d) Planning**  Identify roles and responsibilities, outline specific steps and milestones, and define goals and measures for implementation success in advance. | **Patient Needs and Outcomes** | *I think I had the same goals and ambitions as what they had really, for me to get back to full health and back, you know, back fit again (PA01)* | *You never know, do you, how long your recovery’s going to take and you take it minute by minute, week by week. And I think because I didn’t know at that time how things were going to go, and I was probably too optimistic, yeah, it was just – I felt a little bit blindsided (PA04)* |
|  | **MDT** | *Then we got to a point where we started to talk about my memory, and the concerns of my memory and me, that she thought that obviously the clinical psychologist would probably be better to introduce them at that point. And I can’t remember where that was, but it was a few months into it I think anyway. And then I think we did an introduction session with [the clinical psychologist], where he joined [the occupational therapist] and I, and then from there we went on to [the psychologist] and I having one-to-one sessions, as well as [the occupational therapist] and I having one-to-one sessions, and keeping the two things separated. (PA08)* | *I know they can’t do it as part of the study, but if after the research some kind of screening whereby they work out who actually needs the service and who doesn’t. Or it could be some kind of self-referral, so if someone needs it, they can request it as opposed to necessarily everyone getting a blanket referral. (TA02)* |
|  | **Mentoring** | *I think when they were given that permission to say that, then I think they’ve been quite assertive in being able to say, “No, I can’t take anymore.” And I know it’s meant that some of the sites have had to stop recruiting at different points, but we’ve been able to then counterbalance that by having a longer recruitment period. And I think that’s a better outcome than potentially losing therapists. (ME02)* | *I do think for future studies you need something, like because it’s up to them whether they come or not, but if they don’t turn up then how do you know they’re doing it? And is there any recourse? (ME01)*  *That was one of the things [TA16] and I were speaking about, in the early days, “Oh, it’d be nice if we could do some joint mentoring,” but it just never panned out. (ME02)* |
| **5e) Tailoring strategies**  Choose and operationalize implementation strategies to address barriers, leverage facilitators, and fit context. | **Patient Needs and Outcomes** | *Initially it was small, because there were very small targets and then as my capability, or health improved, being able to, you know, it’s needed more time, which I’ve had plenty of, so they’re obviously good, so the, one of my targets was to build my physical capability and so then that was jogging or walking for an hour, I’d built it up to an hour which I was really pleased with. I wasn’t set an hour target, I was just set ten minutes, and as I was able to do more I continued, because I felt that sense of achievement and then the exercises that she would email me, they’d take maybe an hour, depending how many she sent me, an hour, an hour and a half to two hours, but they wouldn’t, they wouldn’t be done all at once, because of the concentration level, so that would be spread out throughout, and then building concentration in terms of reading, I initially was only doing ten minutes a day, but then was building it up to do a couple of hours a day. (PA02)* | *I said, I didn’t have any flashbacks or nightmares or anything like that, or depression, nothing like that, and physically I managed to get myself back to normal very quickly (PA01)* |
| **5f) Engaging** | **Attract and encourage participation in implementation and/or the innovation.** | | |
| **Innovation Deliverers**  Attract and encourage deliverers to serve on the implementation team and/or to deliver the innovation. | **Mentoring** | *In fact, I think it worked better in a group, because I’ve done mentoring where it’s just one individual, and I think the group format is better than mentoring somebody only on an individual basis. (ME02)* | *So if we’d been able to actually recruit all the OTs and psychologists in the way that we predicted, it would have been nice to have done some profession specific mentoring sessions, and then combined that as a site mentoring. For me, that would have been the ideal. (ME02)* |
| **Recipients**  Attract and encourage recipients to serve on the implementation team and/or participate in the innovation. | **Patient Needs and Outcomes** | *To be honest it didn’t take too much effort on my part because my OT was so approachable and relaxed and it was the way that she communicated…. The fact that it also worked, engaged with the employer is far above my expectations and that’s the real value added because it’s good for me to understand … but if my employer doesn’t understand then it’s kind of, you’re fighting against a losing battle….And the participation with the OT also helped me to help my family understand as well. Because sometimes my family didn’t appreciate, I mean eventually, but then because I looked okay and I was becoming a little bit more active they thought I should be doing more, and that maybe I was being a little bit lazy, and they didn’t quite understand neither that actually that’s something more going off inside. So me having the knowledge and being able to share that and explain that also helped those around me to be a little bit more patient and a bit more supportive. (PA02)* | *I do think she got a bit too dependent because she was starting to be a bit mischievous towards the end, you know like when I say she didn’t want to go back to work and I was like, yeah, there’s something going on here, that’s not right. So, I think she’d like turn the conversation around her agenda with the ROWTATE. “Tell me what I want to hear. I need another two weeks off, yes, okay.” “They said that I need a few more weeks off.” “No, what did you tell them for them to get that opinion?” so it’s two very different things. But I think, in a way, at the end of the day, she had to be ready to go back and me trying to force her before she was wasn’t a good thing and I think it was good that she had still that security and conversation with them and then when she thought, “You know what, I do need to get back,” she still had that safety net of support, easing herself back in, which was crucial. I think that was crucial as well. (CAR01)* |
|  | **Employer Engagement** | *My philosophy on this stuff is, if you rush somebody back into work after one of these things, or because they’re fearful of their terms and conditions package benefits or whatever it might be, it’s a bit of a false economy, because you feel the pain further down the road. So, I think we are very accommodating. (EMP01)* | *The coordination between two occupational health teams, or a handover from one to another to say look, this is now your companies responsibility because all big companies have, you know, most companies have, if that was clearer, I still think we are, I don’t know what is happening in the ROWTATE area now, but in ours, they’re still trying to figure out what to do with him and that drives me nuts as well as him. (EMP03)* |
| **5g) Doing**  Implement in small steps, tests, or cycles of change to trial and cumulatively optimize delivery of the innovation. | **Mentoring** | *I had [CP mentor] through the feasibility trial and then [CP5] taking over in the main trial, so that was really useful experience and the way that both of them ran the sessions, you know, it was very much like facilitated peer support in in many respects, seeing everybody and the diversity of experiences and insights that people have and sort of enabling us to kind of support each other. So, I like kind of their approach as and probably just borrowed a lot from them. But maybe one of the nice things about the mentoring like I would suspect that others who are in the mentoring groups that I'm in would probably be able to serve that function as well for other clinical psychologists who came on board. So, there's that sort of ‘Train the trainer’ model, you know I think the potential there, I think. So, it almost feels like a sort of peer support model sometimes. (ME03)* | *In the early stages, it was a lot of questions were process driven, “How do I do this? How do I do that? When I get to this stage, then what do I do?” So a lot of it was sort of reminding them of, like, “Don’t forget to do the site screening, and the fatigue questionnaire, don’t forget to write a discharge letter and copy it through to the GP.” So it was a lot of those sorts of, “How do I do this? When do I do this?” So process questions. (ME02)* |
| **5h) Reflecting & Evaluating**  Collect and discuss quantitative and qualitative information about the success of implementation. | **Patient Needs and Outcomes** | *I really do hope that everybody, or anybody that’s in a similar position to me, gets the support from you guys. Because it’s critical. It’s a little bit frustrating, this shouldn’t even be a research study, it should already be there, right? (PI02)*  *I think the level of service that we've provided was over and above any expectation that I've ever had …all of it was of key value to me and the decisions that that I made across that year.(EMP06)* | *I think it’s the wrong outcome. (ME01)*  *They have some concerns about whether that what they see will be captured in the outcomes of the trial, because I think sometimes what they would see as growth and therapeutic change for somebody wouldn't necessarily, wouldn't necessarily always be they've returned to work within 12 months of ROWTATE starting so I know that has come up a few times. How we are judged the success of this and will it take into account some people…. A good outcome might be about finding a sense of purpose in many respects and adjusting to their injury, but not necessarily reflected in and they're back at work and or like retaining the same role that they had beforehand. (ME03)* |
| **5i) Adapting**  Modify the innovation and/or the Inner Setting for optimal fit and integration into work processes. | **Remote** | *I think it would’ve been a bit more tiring as well if it was having to go to a physical location, because you would have to get ready, remember you’ve got to be somewhere, you’ve got to plan your journey, you’ve got to plan getting home, so that would be quite overwhelming initially I think. So, but for me it worked really, really, really well. (PA02)* | *we’re trying to build that rapport with people on, like on a virtual platform, which is difficult when you’re used to delivering face-to-face intervention with someone. So I wonder whether that has a bit of an impact as well on their engagement. (TA03)* |
