# Supplementary material for: Implementation barriers and facilitators to a vocational rehabilitation intervention after traumatic injury (ROWTATE) in the UK: qualitative interviews with key stakeholders
Source: BMJ Open. 2026 Jul 30;16(7):e118198. doi: 10.1136/bmjopen-2026-118198 (PMC13422936; doi:10.1136/bmjopen-2026-118198)
Supplement: Supplementary file 4 [file bmjopen-2026-118198.reviewer_comments.pdf]

PEER REVIEW HISTORY

BMJ Open publishes all reviews undertaken for accepted manuscripts. Reviewers are asked to complete a checklist review form and are provided with free text boxes to elaborate on their assessment. These free text comments are reproduced below.

ARTICLE DETAILS

Title (Provisional)

Implementation barriers and facilitators to a vocational rehabilitation service after traumatic injury (ROWTATE) in the UK: Qualitative interviews with key stakeholders

Authors

Mann, Claire; Lindley, Rebecca; Kendrick, Denise; Radford, Kate; Holmes, Jain; Kellezi, Blerina; dasNair, Roshan; Anwar, Fahim; Graham, Laura; Moghaddam, Nima; Crouch, Robert; Hoffman, Karen; Fallon, Steve; Jones, Trevor; Andrews, Isobel; Gibson, Ali; Timmons, Stephen

---

VERSION 1 - REVIEW

**Reviewer**	**1**
**Name**	**Martin, Rachelle A.**
**Affiliation**	**Burwood Academy of Independent Living, Department of Medicine**
**Date**	**15-Mar-2026**
**COI**	**None**

This qualitative implementation study explores barriers and facilitators to implementing the ROWTATE vocational rehabilitation intervention within NHS major trauma services in England. The findings generate practical recommendations for embedding vocational rehabilitation within integrated care systems and scaling hybrid approaches to support return to work after trauma.

This is a well-written and clearly articulated manuscript that contributes appropriately to the broader programme of work describing the development and evaluation of the ROWTATE complex intervention. The study is methodologically coherent and provides a rich implementation-focused perspective supported by a large qualitative dataset and extensive supplementary materials. The inclusion of 80 interviews across a diverse range of stakeholders is a notable strength and provides substantial depth to the analysis. The integration of implementation frameworks, specifically the Consolidated Framework for Implementation Research (CFIR) and the Theoretical Framework of Acceptability (TFA), also strengthens the conceptual grounding of the study and supports a structured interpretation

of the findings. Overall, the manuscript communicates its findings clearly and offers practical insights for vocational rehabilitation service design and implementation. My comments are very minor and relate to clarification of methodological referencing and small issues in the supplementary materials.

Minor comments

1. Clarification of reflexive thematic analysis approach

The manuscript indicates that reflexive thematic analysis was used (p.10). It would be helpful to include a specific reference for the approach adopted (e.g., Braun & Clarke or other relevant methodological sources) to clarify the analytic framework and support methodological transparency.

2. Supplementary materials – interview guides

In the supplementary materials, author comments remain within the interview guide documents and should be removed prior to publication.

Supplementary materials – data analysis table

In the data analysis table (P2 Row 2), it appears that a quotation may be missing.

COREQ checklist

The COREQ checklist included in the supplementary materials would benefit from a clear title to aid navigation and interpretation of the supporting documentation.

Reviewer 2

Name Jones, samantha

Affiliation The University of Manchester, Division of Nursing, Midwifery & Social Work School of Health Sciences Faculty of Biology, Medicine and Health

Date 28-Apr-2026

COI None

Well done on completing study which will bridge the current knowledge. I enjoyed reading this manuscript and have made some suggestions/points to consider which are relatively minor.

Relevance of the Study: Return to work following major trauma has substantial personal, societal, and economic consequences, including long term health and social care costs. This study addresses an important evidence gap by exploring barriers and facilitators to the implementation of major trauma vocational rehabilitation (VR), an intervention that is often inconsistently delivered or poorly embedded in practice. The focus on implementation fac-

tors is particularly valuable, as improving national consistency and equity in VR provision is critical. The findings therefore have clear relevance for policy, service development, and clinical practice.

Abstract: The abstract captures most of the key elements of the study. However, it would benefit from a brief description of the focus and core components of the vocational rehabilitation intervention to help readers unfamiliar with major trauma vocational Rehabilitation understand the context of the findings.

Introduction: The introduction clearly identifies the gap in the evidence regarding implementation of vocational rehabilitation following major trauma. The rationale for the study would be further strengthened by noting that major trauma predominately affects people of working age, thereby reinforcing the significance of vocational return to work interventions.

The introduction is relatively long, and some detail may be more appropriate for the methods section. For example, discussion of recruitment challenges during the COVID 19 pan-demic and detailed descriptions of the intervention procedures could be relocated. The introduction could briefly outline what the intervention is and why it is needed, while detail of the procedures could be described in the methods.

The description of the intervention indicates that occupational therapists assessed patients for psychological need, but what constitutes psychological needs is not clearly defined. It is also unclear how psychological need was assessed consistently across participants, for example were a specific set of questions or a screening tool used. Referral to clinical psychology services is described, yet access to such services is known to vary significantly across major trauma networks, with waiting times often prolonged. It is not clear whether psychological support was consistently available to all participants or whether variation in access may have influenced participant experiences. This represents a potential confounding factor that should be clarified and, if relevant, acknowledged as a limitation.

The study aim is clearly articulated and is appropriately addressed through the selected methodology.

Methods

Data Collection: The description of data collection would benefit from following the chronological order in which the study was conducted. The provision of participant information and the consent process should be described before detailing the semi structured interviews. The description of the interviews (format, topic guide, duration) would also read more clearly if presented in the same section.

Data Analysis: The data analysis section currently lacks sufficient detail to fully understand how systematically the data were analysed. The methods section would be strengthened by:

• Providing a brief explanation of the overarching qualitative analytic approach (e.g. inductive, deductive, or hybrid) and why this approach was most appropriate.

• It would be helpful to explain more clearly how the analysis was carried out. Specifically, it is unclear whether the researchers first identified key ideas directly from what participants said and then linked these ideas to the Theoretical Framework of acceptability (TFA) and Consolidated Framework for Implementation research (CFIR) frameworks, or whether the TFA and CFIR categories were used from the outset to label the data. Clarifying this would help readers better understand how participants' views shaped the findings?

• Explaining whether any data did not align with the TFA or CFIR constructs and, if so, how such data were analysed and incorporated into the findings.

• Clarifying whether reflexive journaling or other strategies were used to support reflexivity and analytic rigour.

Results: If available, providing basic demographic information for patient participants (e.g. age range, employment status, type of injury) would improve the contextual relevance and transferability of the findings.

The selected quotations are well chosen and effectively illustrate the developed themes. Interpretations are clearly grounded in participant data and align well with the analytic framework.

Discussion: The discussion effectively extrapolates the identified themes to inform practical recommendations for implementing vocational rehabilitation in major trauma services. There is a clear and coherent link between reported barriers and facilitators and the com-ponents of VR that should be prioritised in practice.

Future research could explore barriers to recruiting carer participants, as carers play a potentially important but their role in supporting vocational rehabilitation is un-der researched. Limited carer representation may therefore constrain understanding of this aspect of implementation.

As noted earlier, any difficulties or variation in access to clinical psychology services, including waiting times, should be acknowledged as a study limitation, as this may have influenced participant experiences and perceptions of the intervention.

VERSION 1 - AUTHOR RESPONSE

**Reviewer 1**	
This qualitative implementation study explores barriers and facilitators to implementing the ROWTATE vocational rehabilitation intervention within NHS major trauma services in England. The findings generate practical recommendations for embedding vocational rehabilitation within integrated care systems and scaling hybrid approaches to support return to work after trauma.	We thank Reviewer 1 for their positive and constructive feedback on the manuscript and for recognising the contribution of this study to the broader ROWTATE programme of work. We are pleased that the reviewer found the manuscript to be clearly written, methodologically coherent, and of practical relevance. We have addressed the minor comments below.

This is a well-written and clearly articulated manuscript that contributes appropriately to the broader programme of work describing the development and evaluation of the ROWTATE complex intervention. The study is methodologically coherent and provides a rich implementation-focused perspective supported by a large qualitative dataset and extensive supplementary materials. The inclusion of 80 interviews across a diverse range of stakeholders is a notable strength and provides substantial depth to the analysis. The integration of implementation frameworks, specifically the Consolidated Framework for Implementation Research (CFIR) and the Theoretical Framework of Acceptability (TFA), also strengthens the conceptual grounding of the study and supports a structured interpretation of the findings. Overall, the manuscript communicates its findings clearly and offers practical insights for vocational rehabilitation service design and implementation. My comments are very minor and relate to clarification of methodological referencing and small issues in the supplementary materials.	
1. Clarification of reflexive thematic analysis approach<br>The manuscript indicates that reflexive thematic analysis was used (p.10). It would be helpful to include a specific reference for the approach adopted (e.g., Braun & Clarke or other relevant methodological sources) to clarify the analytic framework and support methodological transparency.	Thank you for this suggestion. We have now included a specific reference to Braun and Clarke's reflexive thematic analysis in the Methods section to clarify the analytic approach and enhance methodological transparency.<br>We have added text<br>"…following Braun and Clarke's reflexive approach [52]"
2. Supplementary materials – interview guides<br>In the supplementary materials, author comments remain within the interview guide documents and should be removed prior to publication.<br><br>Supplementary materials – data analysis table<br>In the data analysis table (P2 Row 2), it appears that a quotation may be missing.	We have removed and cleaned of author comments in the interview guides.<br><br>We have added the missing quote.<br><br>[supplementary materials]
COREQ checklist<br>The COREQ checklist included in the supplementary materials would benefit from a clear title to aid navigation and interpretation of the supporting documentation.	Thank you for this suggestion. We have added a clear title to the COREQ checklist in the supplementary materials to aid navigation and interpretation.

	"Supplementary File: COREQ Checklist for Reporting Qualitative Interviews"
**Reviewer 2**	
Well done on completing study which will bridge the current knowledge. I enjoyed reading this manuscript and have made some suggestions/points to consider which are relatively minor.<br>Relevance of the Study: Return to work following major trauma has substantial personal, societal, and economic consequences, including long term health and social care costs. This study addresses an important evidence gap by exploring barriers and facilitators to the implementation of major trauma vocational rehabilitation (VR), an intervention that is often inconsistently delivered or poorly embedded in practice. The focus on implementation fac-tors is particularly valuable, as improving national consistency and equity in VR provision is critical. The findings therefore have clear relevance for policy, service development, and clinical practice.	We thank Reviewer 2 for their positive comments on the manuscript and for their thoughtful suggestions. We are pleased that they found the study enjoyable to read and of value. We have addressed their minor comments in detail below.
Abstract: The abstract captures most of the key elements of the study. However, it would benefit from a brief description of the focus and core components of the vocational rehabilitation intervention to help readers unfamiliar with major trauma vocational Rehabilitation understand the context of the findings.	We have added briefly to the abstract "…included early vocational assessment lasting up to 12 months, case management, employer engagement, and coordinated multidisciplinary support between occupational therapists and clinical psychologists."
Introduction: The introduction clearly identifies the gap in the evidence regarding implementation of vocational rehabilitation following major trauma. The rationale for the study would be further strengthened by noting that major trauma predominately affects people of working age, thereby reinforcing the significance of vocational return to work interventions.<br>The introduction is relatively long, and some detail may be more appropriate for the methods section. For example, discussion of recruitment challenges during the COVID 19 pan-demic and detailed descriptions of the intervention procedures could be relocated.<br>The introduction could briefly outline what the	We have added a note that major trauma predominately affects people of working age, thereby reinforcing the significance of vocational return to work interventions.<br>"and major trauma disproportionately affects individuals of working age"<br><br>We have moved the section on recruitment challenges as requested.<br><br>We have tightened the following section:<br>"The intervention was informed by systematic review evidence, stakeholder engagement, and co-design processes, with mechanisms mapped in a published logic model [7]."

intervention is and why it is needed, while detail of the procedures could be described in the methods.<br>The description of the intervention indicates that occupational therapists assessed patients for psychological need, but what constitutes psychological needs is not clearly defined. It is also unclear how psychological need was assessed consistently across participants, for example were a specific set of questions or a screening tool used. Referral to clinical psychology services is described, yet access to such services is known to vary significantly across major trauma networks, with waiting times often prolonged. It is not clear whether psychological support was consistently available to all participants or whether variation in access may have influenced participant experiences. This represents a potential confounding factor that should be clarified and, if relevant, acknowledged as a limitation.<br>The study aim is clearly articulated and is appropriately addressed through the selected methodology.	We have clarified the assessment and referral process for psychological support and added this detail to the Methods section.<br><br>"In summary, the intervention included a ROWTATE-trained occupational therapist (OT) contacting patients early after their serious injury and providing holistic, personalised support targeting the impact of injury on work. OTs assessed patients' psychological needs as part of a structured holistic assessment of emotional wellbeing (e.g. mood, adjustment, anxiety, and trauma-related responses) and referred to a clinical psychologist (CP) where appropriate. OTs and CPs worked together to address vocational rehabilitation needs, supporting patients to set goals and engage with employers to facilitate a sustainable and monitored return to work for up to 12 months following injury. Access to clinical psychology input varied across sites in line with NHS service provision, which may have influenced participant experiences and is considered in the interpretation of findings."
Methods<br>Data Collection: The description of data collection would benefit from following the chronological order in which the study was conducted. The provision of participant information and the consent process should be described before detailing the semi structured interviews. The description of the interviews (format, topic guide, duration) would also read more clearly if presented in the same section.	We have moved around the content to reflect chronological order as requested.
Data Analysis: The data analysis section currently lacks sufficient detail to fully understand how systematically the data were analysed. The methods section would be strengthened by:<br>• Providing a brief explanation of the overarching qualitative analytic approach (e.g. inductive, deductive, or hybrid) and why this approach was most appropriate.<br>• It would be helpful to explain more clearly how the analysis was carried out. Specifically, it is unclear whether the researchers first identified key ideas directly from what participants said and then linked these ideas to the Theoretical Framework of acceptability	Thank you for these helpful suggestions. We have expanded the Data Analysis section to improve clarity and transparency.<br><br>We have added to this section as follows:<br>To explain the analytical approach<br><br>"A hybrid deductive–inductive approach was taken, combining framework-based coding with reflexive thematic analysis, to enable both theoretically informed and data-driven insights."<br><br>And later in the section<br><br>"…This stage involved a more inductive and interpretive analysis, allowing themes to be

(TFA) and Consolidated Framework for Implementation research (CFIR) frameworks, or whether the TFA and CFIR categories were used from the outset to label the data. Clarifying this would help readers better understand how participants' views shaped the findings?<br>• Explaining whether any data did not align with the TFA or CFIR constructs and, if so, how such data were analysed and incorporated into the findings.<br>• Clarifying whether reflexive journaling or other strategies were used to support reflexivity and analytic rigour.	generated from participants' accounts while drawing on, but not limited to, the organising frameworks."<br><br>To clarify use of frameworks<br><br>"reflecting a deductive approach applied from the outset to organise the data in relation to implementation constructs."<br><br>To clarify data which not align with frameworks<br><br>"Data that did not clearly align with existing TFA or CFIR constructs were coded inductively and retained for further analysis, ensuring that participants' perspectives were not constrained by the framework."<br><br>To clarify reflexive, iterative discussion as part of the analytical process<br><br>"Reflexivity was supported through ongoing analytic discussions within the research team, with attention to how disciplinary perspectives and prior assumptions shaped interpretation."<br><br>We hope these revisions improve clarity.
Results: If available, providing basic demographic information for patient participants (e.g. age range, employment status, type of injury) would improve the contextual relevance and transferability of the findings.<br>The selected quotations are well chosen and effectively illustrate the developed themes. Interpretations are clearly grounded in participant data and align well with the analytic framework.	Thank you for this helpful suggestion. We have now added a participant characteristics table at the start of the Results section, including key demographic and contextual information (e.g. age range, employment status, and injury type where available) to enhance the contextual relevance and transferability of the findings. We are pleased that the reviewer found the quotations and analytic interpretations to be clear and well-grounded in the data.
Discussion: The discussion effectively extrapolates the identified themes to inform practical recommendations for implementing vocational rehabilitation in major trauma services. There is a clear and coherent link between reported barriers and facilitators and the com-ponents of VR that should be prioritised in practice.<br>Future research could explore barriers to recruiting carer participants, as carers play a potentially important but their role in supporting vocational rehabilitation is un-der researched. Limited carer representation may therefore constrain understanding of this	Thank you for these helpful comments.<br>We have now added a statement to the Future Research section highlighting the need to better understand barriers to recruiting and engaging carers.<br>"Future research should also explore barriers to recruiting and engaging carers, whose role in supporting vocational rehabilitation is important but remains under-researched."<br><br>We have also expanded the limitations section to acknowledge variation in access to clinical psychology services.

aspect of implementation.<br>As noted earlier, any difficulties or variation in access to clinical psychology services, including waiting times, should be acknowledged as a study limitation, as this may have influenced participant experiences and perceptions of the intervention.	“In addition, variation in access to clinical psychology services across sites, including differences in availability and waiting times, may have influenced participant experiences of the intervention and should be considered when interpreting the findings.”
